# Unusual cell surfaces, pili, and archaella of *Thermoplasmatales* archaea

**DOI:** 10.1093/ismejo/wraf176

**Published:** 2025-08-13

**Authors:** Matthew C Gaines, Michail N Isupov, Mathew McLaren, Risat Ul Haque, Alejandra Recalde, Rafael Bargiela, Vicki A M Gold, Sonja-Verena Albers, Peter N Golyshin, Olga V Golyshina, Bertram Daum

**Affiliations:** Living Systems Institute, University of Exeter, EX4 4QD Exeter, England, United Kingdom; Department of Biosciences, Faculty of Health and Life Sciences, Stocker Road, EX4 4QD Exeter, England, United Kingdom; Department of Biosciences, Faculty of Health and Life Sciences, Stocker Road, EX4 4QD Exeter, England, United Kingdom; Henry Wellcome Building for Biocatalysis, Department of Biosciences, Faculty of Health and Life Sciences, University of Exeter, EX4 4QD Exeter, England, United Kingdom; Living Systems Institute, University of Exeter, EX4 4QD Exeter, England, United Kingdom; Department of Biosciences, Faculty of Health and Life Sciences, Stocker Road, EX4 4QD Exeter, England, United Kingdom; Living Systems Institute, University of Exeter, EX4 4QD Exeter, England, United Kingdom; Department of Biosciences, Faculty of Health and Life Sciences, Stocker Road, EX4 4QD Exeter, England, United Kingdom; Institute of Biology, Molecular Biology of Archaea, University of Freiburg, 79104 Freiburg, Baden-Württemberg, Germany; School of Environmental and Natural Sciences, Bangor University, Deiniol Rd, Bangor LL57 2UW, Wales, United Kingdom; Living Systems Institute, University of Exeter, EX4 4QD Exeter, England, United Kingdom; Department of Biosciences, Faculty of Health and Life Sciences, Stocker Road, EX4 4QD Exeter, England, United Kingdom; Institute of Biology, Molecular Biology of Archaea, University of Freiburg, 79104 Freiburg, Baden-Württemberg, Germany; Spemann Graduate School of Biology and Medicine, University of Freiburg, 79104 Freiburg, Baden-Württemberg, Germany; Signalling Research Centres BIOSS and CIBBS, Faculty of Biology, University of Freiburg, 79104 Freiburg, Baden-Württemberg, Germany; School of Environmental and Natural Sciences, Bangor University, Deiniol Rd, Bangor LL57 2UW, Wales, United Kingdom; School of Environmental and Natural Sciences, Bangor University, Deiniol Rd, Bangor LL57 2UW, Wales, United Kingdom; Living Systems Institute, University of Exeter, EX4 4QD Exeter, England, United Kingdom; Department of Biosciences, Faculty of Health and Life Sciences, Stocker Road, EX4 4QD Exeter, England, United Kingdom

**Keywords:** Archaea, Thermoplasmatales, Oxyplasma meridianum, Cuniculiplasma divulgatum, cryoEM, cryoET, filaments, archaella, pili

## Abstract

Archaea of the order *Thermoplasmatales* push the boundaries of our current knowledge of prokaryotic life. They show distinct cellular plasticity, heterogenous cell morphologies, and lack a paracrystalline S-layer. As the S-layer has previously been implicated in acting as a stator scaffold for filaments driving cellular propulsion, particularly archaella, we asked whether the absence of an S-layer precludes the formation of functional archaella or pili in *Thermoplasmatales*. Using cryoEM, we investigated the two *Thermoplasmatales* species *Cuniculiplasma divulgatum* and *Oxyplasma meridianum*. We found that these species indeed generate pili and archaella and that the latter likely function in cellular propulsion. Whereas *C. divulgatum* produces pili with terminal hooks using a unique assembly machinery, *O. meridianum* generates unusually wide, “barbed” archaella with a high degree of glycosylation. Our results show that for the generation of functional archaella and pili, a canonical S-layer is not necessary.

## Introduction

Extremophiles have evolved to thrive in conditions such as extreme salinity, acidity, alkalinity, and temperature, which are lethal to mesophilic life forms [1]. Although extremophiles are found in all three domains of life, the majority belong to the archaeal domain. Species of the order *Thermoplasmatales* have a facultative anaerobic or aerobic metabolism, which relies on complex organic carbon substrates. *Thermoplasmatales* species grow at extremely low pH values (down to −0.06 [2]) and temperatures of 37°C–60°C [3].

Archaea are often surrounded by a porous glycoprotein harness known as the surface (S)-layer, which usually forms the outermost component of their cell wall [4–9]. This S-layer serves as their primary protection from harsh environmental conditions, as well as defence against viruses [6]. In addition, the S-layer creates a quasi-periplasmic space between itself and the membrane and acts as a scaffold that anchors specific proteins and molecular machines in the cell envelope [5, 10]. Examples include the archaellum machinery, a rotary swimming propeller analogous to the bacterial flagellum [11], archaeal Type IV pili (T4P), such as archaeal adhesive pili (Aap), which function in adhesion and twitching motility [12, 13], and threads, adhesive filaments similar to bacterial chaperone-usher pili [14].

Archaeal Type IV pilins and archaellins, which form the subunits of pili and archaella, respectively, are expressed as preproteins with a signal peptide. This signal peptide is cleaved in bacteria by the type III prepilin signal peptidase PilD, and in archaea by ArlK/PibD or EppA [15, 16], resulting in mature pilin proteins. The pilins/archaellins are then assembled into a filament, wherein the hydrophobic alpha helical N-termini are helically stacked in the core of the filament, and the hydrophilic β-strand-rich heads are oriented towards the filament's periphery [17].

The assembly of T4P is driven through ATPases that form a central component of larger molecular machines. The widespread bacterial Type IVa pili (T4aP), as opposed to T4bP that are exclusive to enteric bacteria, provide twitching motility, and function with the aid of a retraction ATPase [18]. In contrast, archaeal T4P typically do not have retractive ATPases within their operons [19]. Moreover, the archaeal T4P machinery typically contains fewer genes than that of Gram-negative bacteria. Due to the lack of an outer membrane in most archaea, these species have no need for peptidoglycan-resolving or outer membrane conduit (secretin) proteins. In the archaeon *Sulfolobus acidocaldarius*, the Aap machinery is thought to consist of at least five proteins; the main filament-forming protein (AapB), a minor pilin (AapA), the cytoplasmic hexameric ATPase (AapE), a membrane-bound, likely dimeric assembly platform protein homologous to the bacterial T4P platform PilC (AapF), and a protein with unknown function called AapX. The latter is predicted to be involved in surface binding through sugar/iron binding capabilities [12, 20].

The archaellum machinery has been studied in greater detail. It consists of a membrane-embedded multi-protein basal body that drives the assembly and rotation of the ArlB filament [21]. Like T4P, the archaellum machinery is powered by a hexameric motor ATPase (ArlI), which is located in the cytosol. ArlI hydrolyses ATP to initially power the assembly of the archaellum filament and, putatively by the action of ArlH, switches to filament gyration, once archaellum assembly is complete [22, 23]. The membrane-bound platform protein ArlJ is thought to be a dimer, spanning the cell membrane, and interacting with ArlI in the cytosol [21]. ATP hydrolysis by ArlI is likely caused by conformational changes in ArlJ, which hoist ArlB subunits from the membrane and integrate them into the nascent filament. In the pseudoperiplasmic space, filaments of ArlG are believed to span from the cellular membrane to the S-layer. Capped by ArlF proteins, an interaction between the ArlFG complex and the paracrystalline S-layer array is proposed to be formed [24, 25]. *Thermoproteota (e.g. Sulfolobus species)* contain a ring of ArlX proteins [26], thought to encase the ArlH/ArlI complex and to integrate it into the cellular membrane. In many *Methanobacteriati* (such as *Pyrococcus furiosus* and *Haloferax volcanii)*, ArlC and ArlD/E have been suggested to replace ArlX [21] and to connect the archaellum with the chemoreceptor machinery [17, 27].

It has been postulated that the stator subunits ArlG and ArlF anchor the rotary motors in the S-layer and thus prevent their futile rotation in the membrane [25]. This is supported by the observation that mutant strains of *S. islandicus* that lack S-layers are immotile, even though they still assemble archaella [24]. Moreover, it has recently been shown that some archaea are capable of twitching motility through cycles of extending and retracting pili, called Aap [28]. It is conceivable that the S-layer also plays a role as a scaffold for pilus assembly and disassembly. Nevertheless, most *Thermoplasmatales* species do not appear to possess a canonical S-layer [29, 30] but a distinctive membrane instead, which is primarily comprised of a tetraether lipoglycan [31, 32]. This unique lipoglycan is characterized by an atypical archaeal tetraether lipid linked to an oligosaccharide, which is composed of glucose and mannose [33]. It is postulated that this lipoglycan plays a pivotal role in conferring acid and thermal stability in the membrane of members of the *Thermoplasmatales* [32, 34]. The scarcity of cultivated *Thermoplasmatales* species has so far been a limiting factor in the study of their cell biology. Thus, little is known about their cellular morphology or the architecture of their cell envelope. Whereas some species appear to encode T4P or archaella in their genomes, it is unclear if these are indeed assembled, and if these species are motile via twitching or swimming propulsion.

In this study, we investigated the recently discovered *Thermoplasmatales* species, *Oxyplasma meridianum*, and the previously described *Cuniculiplasma divulgatum* [35–37]. By examining their morphology using electron cryo-tomography (cryoET), we confirm that neither *C. divulgatum* nor *O. meridianum* possesses a *bona fide* S-layer array but instead a dense layer of largely unordered proteins. Moreover, we show that both species extend surface filaments, confirming previous observations in the *Thermoplasmatales* species *Thermoplasma volcanium* [38]. Through single particle cryo-electron microscopy (cryoEM) and helical reconstruction, we solved the structures of these filaments at 2.61 and 2.52 Å resolution for *C. divulgatum* and *O. meridianum,* respectively. Atomic model building, bioinformatics analysis, and motility experiments suggest that the *C. divulgatum* fibre is a nonrotary, likely adhesive T4P, whereas the *O. meridianum* filament is an archaellum. Our motility experiments confirm that *O. meridianum* cells are indeed capable of swimming motility, despite the absence of a canonical para-crystalline S-layer.

## Materials and Methods

### Cell growth and filament isolation

For cultivation of strains *O. meridianum* M1^T^ (DSM 116605^T^) [37], and *C. divulgatum* S5^T^ (JCM 30642^T^) [35], the modified medium DSMZ 88 was used, which contained (g l^−1^): (NH_4_)_2_SO_4_, 1.3; KH_2_PO_4_, 0.28; MgSO_4_ .7H_2_O, 0.25; CaCl_2_.2H_2_O, 0.07; FeCl_3_.6H_2_O, 0.02. Trace element solution SL-10 (DSMZ medium 320) in proportion 1:1,000, betaine 0.06% w/v), vitamin solution by Kao and Michayluk (Product N K3129, Sigma-Aldrich, Gillingham, UK) at 1:100 (v/v) were also added. The medium was supplemented with Beef Extract and Tryptone (both Sigma-Aldrich, Gillingham, UK), at a final concentration of 1 g l^−1^ for cultivation of M1, and with beef extract only at a final concentration of 3 g l^−1^ for cultivation of S5 strain, correspondingly. The pH of the medium was adjusted to pH 1.0 by 10 N H_2_SO_4_. The cells of both organisms grew in triplicates, in 250 ml Erlenmeyer flasks containing 50 ml of media at 40°C in a shaking incubator at 100 rpm. The growth was monitored by measurement of optical density at the wavelength 600 nm in a BioPhotometer Plus (Eppendorf, Hamburg, Germany).

These 40 ml cultures were then centrifuged at 5,000 × *g* for 30 min twice to pellet whole cells. The supernatant was then transferred to ultra-centrifuge tubes and centrifuged at 42, 000 rpm for 90 min. This pellet contained sheared filaments and was subsequently resuspended in 100 μl of the supernatant. The samples were stored at 4°C.

### Messenger RNA sequencing

*Oxyplasma meridianum* M1 and *C. divulgatum* S5 were sampled at mid-exponential (day 3 of cultivation), as well as early stationary (day 5) growth phases for light microscopy and RNA extraction. RNA was extracted using PureLink RNA Mini kit, and concentrations were measured by Qubit RNA BR Assay Kit (Invitrogen, ThermoFisher Scientific, Paisley, UK). mRNA sequencing was done using a NovoSeq 6000 System (Illumina) with 150 PE sequencing at Biomarker Technologies (BMK) GmbH (Münster, Germany), alongside bioinformatics analysis. RNA sequencing data are deposited to Gene Expression Omnibus (GEO) at NCBI under accession numbers GSE246948 for *C. divulgatum* and GSE246949 for *O. meridianum*.

### Negative stain transmission electron microscopy

Five-microlitre suspensions of *O. meridianum* and *C. divulgatum* cells were applied to freshly glow-discharged 300 mesh carbon-coated copper grids (Plano GmbH, Wetzlar, Germany), which were then incubated for 30 s. Excess liquid sample was removed via blotting and grids were washed once with 3 μl water. This was repeated three times. The grids were then stained with 2% uranyl acetate. A 120 kV Tecnai Spirit microscope (Themo Fisher Scientific), equipped with a OneView CMOS detector (Gatan), was used to image the samples. No gold fiducials were used in the sample preparation.

### Electron cryo-tomography

Tomograms were recorded using a Titan Krios TEM (Thermo Fisher Scientific) operated at 300 kV. The Krios was equipped with an energy filter and Gatan K3 direct electron detector (Gatan Inc., Pleasanton, USA), running in counting mode. Tilt series were collected in a dose-symmetric fashion, using the EPU software (Thermo Fisher Scientific). A tilt range of max. −60° to +60° and increments of 3° were applied. The magnification was set to 40,000×, resulting in a final pixel size of ~4.6 Å. A defocus of 6–8 μm was applied. Tilt series were recorded in dose-fractionation mode at a dose rate of 10–12 e^−^ per pixel per second, six fractions per tilt, and with a total dose of 120 e^−^/Å^−2^. Motion correction and CTF estimation of movies were performed using Warp [39]. Tilt series were aligned in AreTomo 2.0 [40], and returned to Warp, where CTF-corrected and deconvolved tomograms were reconstructed. Tomograms were displayed as solid surfaces in ChimeraX 1.9 [41] and coloured by radius with the cell’s centre as origin, using the surface colour function in ChimeraX.

### Cryo-EM sample preparation and data collection

3 μl suspensions of *O. meridianum* and *C. divulgatum* filaments were pipetted onto glow-discharged 300 mesh copper R2/2 Quantifoil grids. 597 Whatman filter papers were used to blot the grids for 5 s, combined with a blot force of −1, at 95% relative humidity, and 21°C. Plunge freezing into liquid ethane was performed using a Mark IV Vitrobot (Thermo Fisher Scientific). The grids were then screened using a 120 kV FEI Tecnai Spirit EM, combined with a OneView CMOS detector (Gatan).

For high-resolution image data collection, a 200 kV Talos Arctica electron microscope (Thermo Fisher Scientific), combined with a Gatan K2 Summit electron detector (Gatan), operating in counting mode was used. Data were collected at a calibrated magnification of ×130,000 (relating to a pixel size value of 1.05 Å). The EPU software package (Thermo Fisher Scientific) was used to control the camera. Movies were recorded at a dosage of 10.6 e^−^/Å^2^s at 50 fractions per 5 s exposure, with an accumulated total dose of 53 e^−^/Å^2^, and a set defocus range of −0.8 to −2 μm, using 0.4 μm steps. Cryo-EM statistics are shown in Supplementary Table 1.

### Single particle image processing

The cryoSPARC [42] full-frame motion correction package was used to align the frames of 4,130 movies for *O. meridianum* and 3,159 movies for *C. divulgatum*. Defocus variation was then estimated for both datasets, using the cryoSPARC patch CTF estimation programme [42]. The native picker within cryoSPARC [42] was used to manually pick filaments for both datasets. These templates were then implemented into the Filament Tracer programme, which successfully traced filaments for both datasets. From here, iterative rounds of 2D classification were performed for each dataset to select high-quality particles.

A total of 1,229,751 and 645,543 helical segments were selected for *O. meridianum* and *C. divulgatum*, respectively. These were then subjected to non-biased 3D refinement, from which initial helical parameters were deduced (~5 Å rise and ~105° twist). Through helical symmetry search, the helical parameters were then refined to 5.65 Å rise and 107.992° twist for the *O. meridianum* archaellum, and 5.18 Å rise and 106.047° twist for the *C. divulgatum* filament. Global and local CTF refinements, as well as reference-based motion correction were carried out before final helical refinement jobs, ultimately leading to global resolutions of 2.5 Å and 2.6 Å for *O. meridianum* and *C. divulgatum,* respectively. These resolutions were estimated using Fourier shell correlation between two independently refined half sets, using the gold-standard value of 0.143. Local resolutions were calculated within cryoSPARC [42] and maps were visualized in ChimeraX [41].

### Atomic model building and validation

ModelAngelo [43] was used to generate initial atomic models. The sequences of the resulting (incomplete) models were then used for genome-wide searches. Based on sequence length, glycosylation sequon locations and large residue positions, the subunit proteins were identified as OXI_ME_000412 for *O. meridianum* and CSP5_RS05915 for *C. divulgatum*. These findings were further corroborated through AlphaFold2 [44]. Manual initial model building was performed in Coot [45], before MOLREP [46] was used for phased molecular replacement, and to position the remaining monomers into the density. CCP4 [47] was used to refine the built chains. Coot was used to model the glycan structures from *T. acidophilum* [48], with the unusual sugar dictionary being prepared using the JLIGAND [49]. The final structure was refined using REFMAC5 via the ccpem interface [50, 51].

### Sequence analysis and structural prediction

The major pilin sequences of both filaments were loaded into SyntTax [52] to search for homologous sequences amongst other *Thermoplasmatales* species. The only two results found for *O. meridianum* were then compared against each other using Clustal Omega [53]. The Kyoto Encyclopedia of Genes and Genomes (KEGG) database [54] was employed to locate the operons in the various homologous species described. FlaFind [16] and SignalP6.0 [55] servers were used to predict signal sequences.

### Motility assays in semisolid gelrite plates

Motility plates were prepared using the modified Medium 88 at pH 1–1.5, supplemented with 0.06% betaine, Kao and Michayluk vitamin solution (1:100), beef extract and tryptone (0.0025% each), and 0.25% gelrite (Carl Roth, Karlsruhe, Germany). The plates were incubated for 14 days at 40°C and 45°C in a humidity chamber and were documented on Days 4 and 14 using an Epson Perfection V800 scanner. The swimming area was calculated for each spot using Fiji [56], and the mean was graphically analysed for both days. The corresponding figures were prepared in Prism (GraphPad) and Adobe Illustrator.

### Swimming and twitching analysis by live microscopy

*Oxyplasma meridianum* cells were grown to the exponential stage (OD_600_ 0.4–0.5). Subsequently, cells were diluted in prewarmed media and transferred to a 0.17 mm Delta T dish (Bioptechs). For imaging, a phase contrast Axio Observer Z1, Zeiss microscope equipped with a Plan-Apochromat 100× 1.40 Oil PH3 M27 objective (NA: 1.4), and a temperature-controlled chamber was used. Swimming cells were observed at 40°C or 55°C. For the imaging at 55°C, cells were incubated at that temperature for 1 h, after reaching an exponential phase OD_600_ at 40°C. Time-lapse movies of 30 s to 1 min were acquired, recording continuously on streamer mode, and were subsequently processed with the Zen software.

Twitching motility was analysed for *C. divulgatum* at two different OD_600_ values (0.3 and 0.5), using the controlled temperature VAHeat device (Interherence GmbH, Germany) heated to 40°C, and Axio Observer Z1 Zeiss microscope equipped with Plan-Apochromat 100× 1.40 Oil DIC M27 objective (NA: 1.4). Movies were recorded at 1 frame/s for 5 min. Analysis of twitching was done in Fiji [56], using the trainable Weka detector [57]. A movie with 20 frames and 600 × 600 pixels was used to train the detector to select cells in focus (attached to the surface). The model was employed in TrackMate7 [58] to track the cells. After defining the threshold for cell radius, shape index, and track length, the tracks were refined by visual inspection and manual pruning.

## Results

### *C. divulgatum* and *O. meridianum* display an unordered protein shell instead of an S-layer

**Figure 1:**
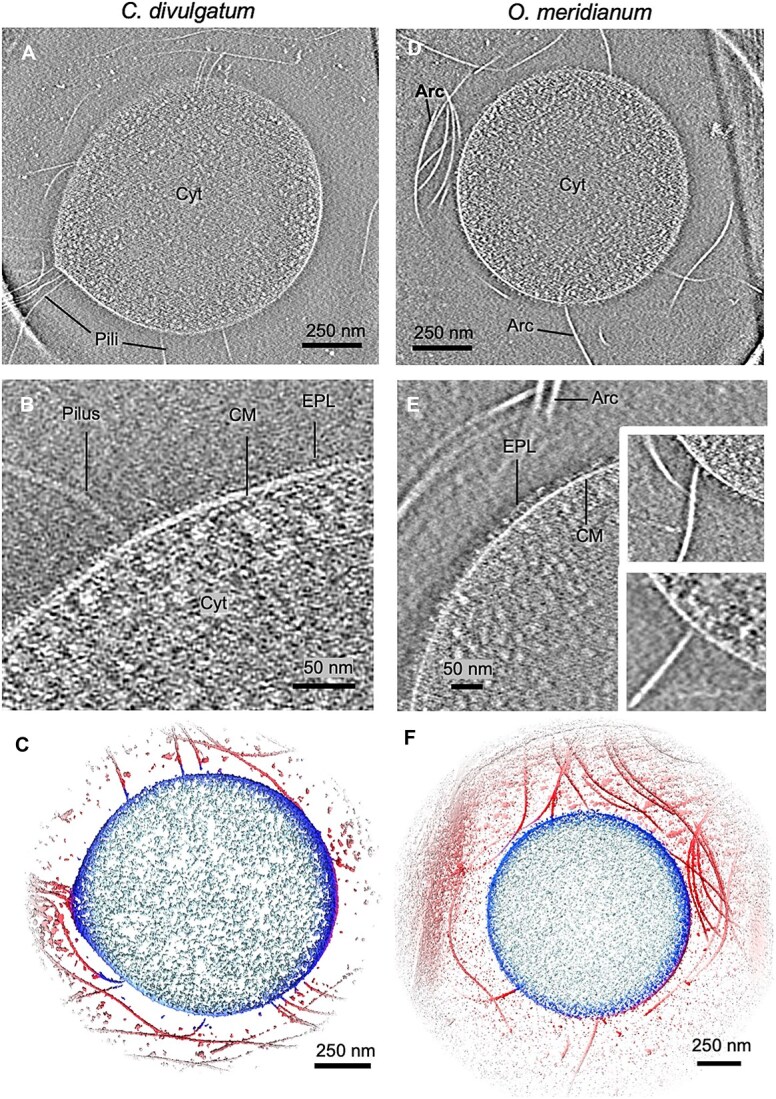
Cell morphology of *C. divulgatum* and *O. meridianum.* (A, D) Tomographic slices of *C. divulgatum* (A), *O. meridianum* cells (D), and closeups of the cell surface (B, E). CM, cell membrane; EPL, exterior protein layer; Cyt, cytosol; Arc, archaellum. Insets in (E) show attachment points for archaella in the cell surface of *O. meridianum.* (C, F). Coloured isosurfaces showing *C. divulgatum* (C) and *O. meridianum* (F) cells in 3D. Light blue, cytoplasm; dark blue, membrane; red, filaments and other extracellular densities.

Cryo-tomograms of *O. meridianum* and *C. divulgatum* grown to mid-exponential phase revealed that the cells were mainly coccoid in shape. Cell diameters varied from 700 to 1000 nm for *C. divulgatum,* and 800 nm to 1500 nm for *O. meridianum*. The cells displayed an average *z*-dimension (thickness) of 290.1 nm (stdev 60.9 nm) for *C. divulgatum* and 243.7 nm (stdev 69.1 nm) for *O. meridianum.* This reduced *z*-dimension is likely due to the cells being “squeezed” by surface tension forces in the vitreous suspension that arise during the blotting step of cryoEM sample preparation. This can lead to an artificial expansion of the *x*/*y* dimensions, suggesting that the true diameter of the roughly spherical cells would be significantly smaller. High-pressure freezing followed by Scanning Transmission Electron Microscopy (STEM) or Focused Ion Beam Scanning Electron Microscopy (FIB-SEM) would be required to obtain closer to native measurements of the cell morphology. Nevertheless, we did not observe a para-crystalline S-layer that typically surrounds other archaea, such as *P*.* furiosus* [59]*, H*.* volcanii* [60]*, or Sulfolobus* species [61]*,* thus confirming previous morphological studies [35, 36]. Accordingly, genome-wide searches did not uncover any S-layer-like genes in either *C. divulgatum* or *O. meridianum*. Instead, we observed a dense, ~20 nm-thick layer of proteins on the outer surface of the cell membrane of both species, which did not appear to form a regular lattice (Fig. 1b and e). We will henceforth refer to this layer as the exterior protein layer (EPL). In addition, the tomograms revealed that both species extended long, undulating surface filaments (Fig. 1).

### *O. meridianum* and *C. divulgatum* possess different types of filaments

Culture samples collected at mid-log phase and early stationary phase investigated by negative stain EM revealed that the surface filaments of *C. divulgatum* and *O. meridianum* have distinct features (Supplementary Fig. 1–3). *Cuniculiplasma divulgatum* generates large quantities of filaments measuring ∼10 nm in diameter (Supplementary Figs 1 and 2). The filament appears to be highly flexible, reminiscent of archaeal adhesive pili (Aap) found in *Sulfolobales* species [12, 13]. In contrast to Aap, the *C. divulgatum* filaments showed clear hook-like structures at their cell-distal termini. Each hook appeared to have a smaller diameter than the main filament and emerged from it in an obtuse angle (Supplementary Fig. 2D). *Oxyplasma meridianum* produces several filaments with 13 nm diameter per cell (Supplementary Fig. 1). These filaments measure a few micrometres in length, undulate in a superhelical fashion, and have rather blunt ends (Supplementary Fig. 3), akin to archaella [25, 62, 63].

### High-resolution cryoEM of the *C. divulgatum* and *O. meridianum* filaments

To unambiguously identify the distinct filaments from *C. divulgatum* and *O. meridianum* and gain insights into their structure, we grew cell cultures of both species to early stationary phase. We then harvested the filaments by differential centrifugation, recorded cryoEM data using a 200 kV Talos Arctica microscope, and reconstructed the structures of the filaments using the CryoSPARC software package [42] (Supplementary Table 1). After cleaning the dataset by iterative 2D classifications, the Helix Refine programme was employed to generate nonbiased 3D reconstructions for both filaments without applying helical parameters [42]. In those maps, filament monomers were resolved, and helical parameters could be deduced in real space as published previously [12, 14].

**Figure 2:**
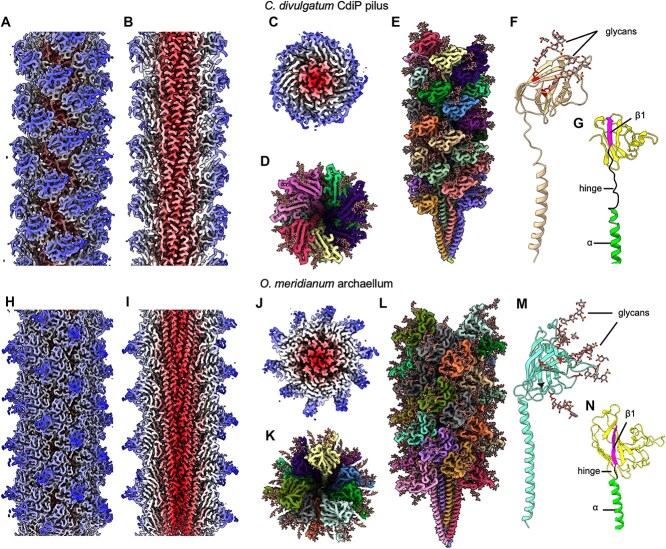
Structures of the *C. divulgatum* pilus and the *O. meridianum* archaellum. (A–C) cryoEM map of the *C. divulgatum* pilus in side view (A), cross section (B), and end-on view (C). The map is colored by radius (core ⍺-helices in red and peripheral globular β-strand rich domains in white and blue). (D, E) atomic model of the pilus in end-on (D) and side view (E), colored by subunit. The protein backbone is shown in liquorice and glycans in stick representation. (F) atomic model of a single *C. divulgatum* pilin. Protein is shown as ribbons and glycans as sticks, colored by element. (G) pilin colored by domain. N-terminal ⍺-helix is green, hinge is black, β1 is pink, and head is yellow. For simplicity, glycans were omitted. (H–J) cryoEM map of the *O. meridianum* archaellum in side view (H), cross section (I), and end-on view (J). The map is colored by radius (core ⍺-helices in red and peripheral globular β-strand rich domains in white and blue). (K, L) atomic model of the archaellum in end-on (K) and side view (L), colored by subunit. The protein backbone is in liquorice and glycans in stick representation, coloured by element. (M) Atomic model of a single *O. meridianum* archaellin. Protein is shown as ribbons and glycan as sticks, colored by element. (N) archaellin colored by domain with glycans omitted for simplicity. The N-terminal ⍺-helix is green, the hinge is black, β1 is pink, and the head is colored yellow.

The helical parameters were determined as ~5 Å rise and ~105° twist, in line with previously investigated archaella and Aap [59, 64, 65]. Based on these initial parameters, we performed helical symmetry searches that suggested refined symmetry operators (−5.18 Å rise and 106.047° twist for *C. divulgatum*, and 5.65 Å rise and 107.992° for *O. meridianum*). Applying these values resulted in maps with global resolutions of 2.8 and 2.6 Å, respectively. The resolution values further improved to 2.6 and 2.5 Å through local CTF refinement and polishing (Supplementary Table 1). Local resolution estimation revealed a resolution of 2.3–2.4 Å at the core and 3.3 Å–3.4 Å at the periphery of both filaments (Supplementary Fig. 4). The final cryoEM maps exhibited key features of T4P-like filaments [12, 59, 62, 64, 66], with a central ⍺-helical core, and an outer sheath composed of β-strand-rich globular domains (Fig. 2a–c and h–j).

### Filament identification

After obtaining high-resolution maps of both filaments by cryoEM, we used ModelAngelo [43] to automatically build atomic models and thus identify the sequences of their constituting subunits. Although the resulting models were incomplete, they both contained sufficient information to blast the sequences against the known genomes of *C. divulgatum* and *O. meridianum.* The locations of glycosylation sites in our density map, the predicted gene length, as well as key residues predicted by ModelAngelo [43], allowed for an unambiguous identification of the corresponding genes (Supplementary Fig. 5).

**Figure 3:**
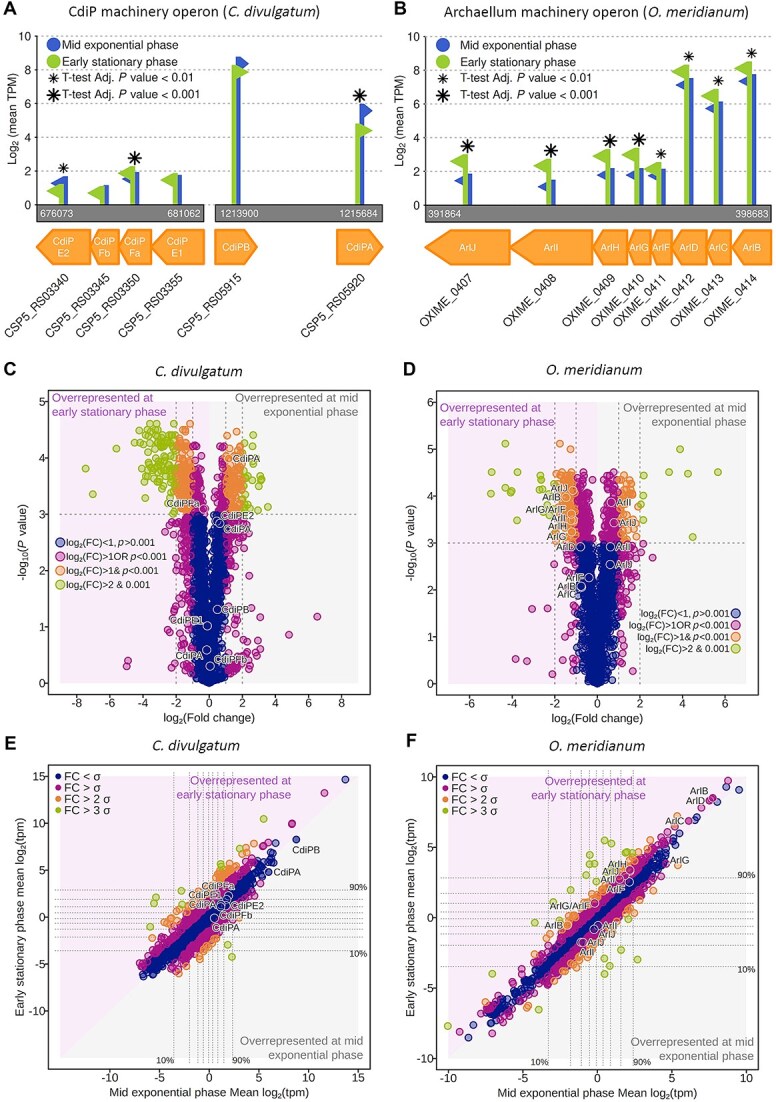
Transcriptomics for *C. divulgatum* and *O. meridianum* in mid exponential and early stationary growth phases. (A, B) mRNAseq data for the *C. divulgatum* CdiP pilus machinery (A) and *O. meridianum* archaellum machinery (B) operons. Genes are depicted in orange; blue indicates expression levels at mid exponential phase, and green indicates expression at early stationary phase. (C, D) Volcano plots showing differential mRNA expression of key genes in *C. divulgatum* (C) and *O. meridianum* (D) in mid-exponential and early stationary growth phases**.** The plots show the log2 (fold change) (FC) difference between mid-exponential and stationary phase, and *t*-test *P*-values corresponding to each locus. Difference is colour coded according to the log2 (fold change) difference (<1-, >1-, or 2-fold and >2- or 4-fold) of the *P*-value (> or < to 0.001). OXIME_000405–000412 refer to the *O. meridianum* archaellum operon genes *arlJ*, *arlI*, *arlH*, *arlG*, *arlF*, *arlD*, *arlC*, and *arlB*. (E, F) Transcript abundance based on transcripts per million (tpm) of each locus for *C. divulgatum* (E) and *O. meridianum* (F), comparing mid-exponential and early stationary growth phases. Colours are based on the difference between each locus log2 (fold change), as well as the standard deviation among all log2 (fold change) of all loci. Dotted lines represent the loci accumulation at each 10 percentile. Labels in both graphics correspond to the locus tag of surface-related protein.

The subunit for the *C. divulgatum* was identified as CSP5_RS05915 (Supplementary Fig. 6A). mRNA sequencing (RNAseq) showed that this gene is highly transcribed (Fig. 3A) and resides in a gene cluster similar to that encoding the Aap machinery in *Sulfolobales* [12] (Fig. 3A; Supplementary Fig. 6A and B). The *O. meridianum* filament subunit gene (OXIME-000412) showed similarly high expression levels (Fig. 3B) and is encoded in a gene cluster typical for archaella found in species of the phylum *Methanobacteriota* (containing AlrC and ArlD homologues) (Supplementary Fig. 7A and B) [67]. Comparing our experimental structures with Alphafold2 [44], predictions resulted in close matches, further confirming the correct identification of the subunits (Supplementary Fig. 8). We will henceforth name the filament of *C. divulgatum* pilus (CdiP), and, by applying the same nomenclature system as for the genes of the homologous *S. acidocaldarius*, we will refer to its subunit pilin as CdiPB (Fig. 3A, Supplementary Fig. 6).

RNAseq data of *C. divulgatum* indicated that CdiPB is the most expressed gene of the operon (Fig. 3A) and indeed in the entire cell, which is consistent with the large number of pili observed (Supplementary Fig. 1C and D). Another predicted pilin that is encoded adjacent to CdiPB is expressed at far lower levels (Fig. 3A, C, E). Alphafold2 [44], predictions of the gene product of CdiPA resulted in a structure incompatible with our cryoEM map of the pilus (Supplementary Fig. 8C). Similar to AapA of *S. acidocaldarius*, CdiPA may thus function as a minor pilin—perhaps as a part of the assembly machinery.

The subunit for the *O. meridianum* filament was identified as OXIME_000412 (Fig. 2K–N). No other archaellin candidates were found in the operon (Fig. 3B, Supplementary Fig. 7). This ruled out a mixed population of subunits in the archaellum, as seen in Methanocaldococcus villosus [62]. In accordance with the nomenclature for archaellins of the phylum *Methanobacteriota*, we will name the archaellin ArlB and, consequently, the archaellum machinery proteins encoded in the operon ArlC, D, F, G, H, I, and J (Fig. 3D, Supplementary Fig. 7).

### Structure of the CdiP pilus

**Figure 4:**
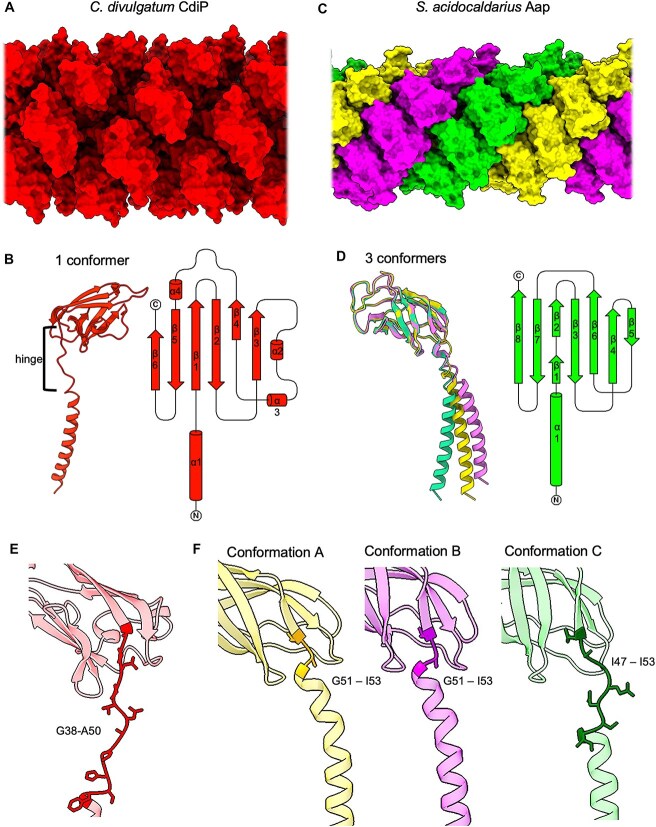
Comparison between CdiP and Aap pili. (A) Structure of the CdiP pilus in surface representation. The pilus consists of the pilin CdiPB, which is shown in ribbon representation in (B, left) and as a fold diagram (B, right). (C) Structure of the *S. *a*cidocaldarius* Aap (PDB-8Q30 [12]) in surface representation. The filament (C) consists of AapB (D), which adopts three conformations (yellow, magenta, green). In D, AapB is shown in ribbon representation (left) and as a fold diagram (right). The relative positions of the three conformations within the Aap pilus are shown in C. In contrast, CdiPB does not adopt three distinct conformations in the pilus (A, B). In contrast to AapB, the head domain of CdiPB includes additional ⍺-helices (⍺2, ⍺3, ⍺4), as well as a loop between β4 and ⍺4 (B, D). CpdB (E) and AapB (F) have different hinge regions between their N-terminal tails and C-terminal heads. (E) CdiPB has one particularly long hinge, which reaches from G38 to A50. (F) In contrast, the hinge in AapA changes, dependent on its conformation. Conformations A and B show short hinges, including residues G51 – I53. Conformation C has a larger hinge, spanning between residues I47 – I53.

Archaeal and bacterial T4P-like filaments have a common ancestry [15, 19] and thus overall similar structures. Whereas the N-terminal ⍺-helices are highly conserved, the globular C-terminal domains vary to adopt different surface properties. This likely adapts species to their unique ecological niches. CdiPB abides by this T4P blueprint [68, 69] (Figs 2F and G and 4B). The protein is 135 residues long and folds into an N-terminal α-helical tail, followed by a globular head. The first 11 amino acids of the genome-encoded sequence are missing in the mature protein. The FlaFind server [16], determined a class-III peptidase cleavage site between residues 11 and 12, indicating that the first 11 residues are proteolytically removed during the maturation of the protein, as is typical for Type-IV pilins The head domain is composed of six β-strands that fold into two β-sheets but unusually also contains three short α-helices (α2, α3, and α4; Fig. 4B). In addition, CdiPB contains a comparatively long linker region of 12 amino acids between the head and the tail of the protein (G38 to A50; Figs 2G and 4B and E).

We showed previously that the Aap of *S. acidocaldarius* has a complex tri-helical superstructure (Fig. 4C) [12]. In each of its three component helices, AapB adopts a unique conformation, distinguished by the angle between the head and the tail domain, as well as the structure of the hinge (Fig. 4D and F) [12, 13]. Such a three-conformer architecture is not evident in the CdiP pilus (Fig. 4A, B, E).

When building the atomic model for CdiPB, we identified two N-linked glycans for each subunit, originating from conserved NXS/T sequons (N64 and N106; Fig. 2F, Supplementary Fig. 9A–C) [14, 59, 62]. As the exact glycan structure of *C. divulgatum* is not known, we modelled the glycan of the closely related *T*.* acidophilum* [48], to visualize the distribution of the glycans over the filaments. The densities for the two glycosylation sites were well resolved, allowing the assignment of six and five sugars for N64 and N106, respectively (Supplementary Fig. 9A and B). The connectivity and chirality of the *T. acidophilum* sugars matched the observed density in CdiPB well. Therefore, the glycans were modelled and glycan dictionaries generated for the uncommon sugars components [D-glycero-D-galacto-heptose (Hep) and 6-deoxy-6-C-sulfo-D-galactose (Fuc6S)] (Supplementary Fig. 9A and B).

### Structure of the *O. meridianum* archaellum

The ArlB of *O. meridianum* is largely a typical archaellin, consisting of an N-terminal α-helical tail and a globular head domain (Fig. 2M and N). The *O. meridianum* pre-archaellin ArlB has a length of 254 AA, of which the 26 most N-terminal residues form the leader peptide. This leader sequence is unusually long compared to archaellins of other archaeal species (11 for *S. acidocaldarius* and 5 for *P. furiosus*).

**Figure 5:**
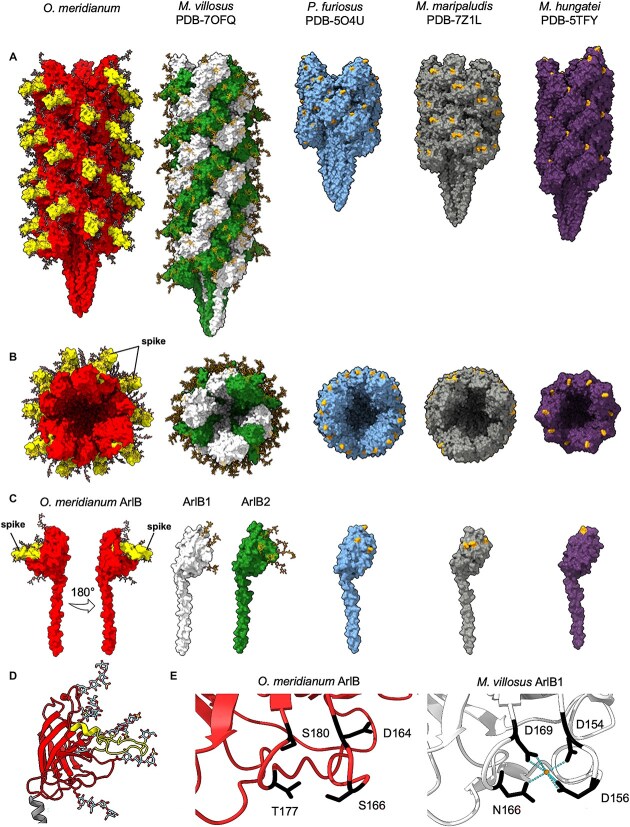
Structure of the *O. meridianum* archaellum in comparison with known archaella from *Methanobacteriati*. Structure of the *O. meridianum* archaellum compared to those of *Methanobacteriati* archaella in side (A) and end-on views (B). (C) The structures of the *O. meridianum* archaellin compared to those of the archaella from *Methanobacteriati* in (B) and (C). *O. meridianum* (red, with barbes in yellow), *M. *v*illosus* (white and green for ArlB1 and ArlB2, respectively; PDB-7OFQ [62]), *P. *f*uriosus* (blue; PDB-5O4U [59]), *M. *m*aripaludis* (grey; PDB-5Z1L [64]), and *M. *h*ungatei* (purple; 5TFY [66]). (D) Model of the *O. meridianum* archaellin ArlB, showing the α-helical N-terminal domain in grey, the globular head domain in red, and the barb domain in yellow. Glycans are shown in stick representation. (E) Metal ion coordination site in the *M. *v*illosus* archellin ArlB1 (white), compared to the same site in AlrB of *O. meridianum*. The *M. villosus* archaellin features a Ca^2+^ coordination site, which is absent in *O.meridianum.*

The *O. meridianum* archaellum appears to be the widest of all so far structurally investigated archaella (130 Å, compared to, e.g. 105 Å for *M. villosus* [62] and 90 Å for *M. hungatei* [66], (Fig. 5A and B). The additional width is due to a prominent spike-like subdomain (V126 – L153) in the *O. meridianum* ArlB protein, which is absent in other archaellins (Fig. 5A–D). This spike domain consists of a two-turn ⍺ helix and an extended loop of 22 amino acids, interrupted by a second short helix (Fig. 5D). In the assembled filament, these spikes give the archaellum a barbed surface profile.

*Oxyplasma meridianum* ArlB boasts eight glycans (N62, N72, N91, N113, N121, N138, N150, N187; Figs 2M and 4D), making it the most heavily glycosylated archaeal filament protein known to date. As for the *C. divulgatum* pilus, we modelled the *T*.* acidophilum* glycan tree [48] into the corresponding densities, which fitted the data well (Supplementary Fig. 9D–F). Nine N-glycosylation sequons are present in total; however, N206 faces inside the β-sheet head and thus is likely inaccessible to posttranslational modification. Only two of the eight glycans are bound to the spike domain, suggesting that it has not evolved to serve as a dedicated glycosylation sub-domain, as seen in the archaellum of *S. acidocaldarius* [67].

Some archaella coordinate divalent ions (Ca^2+^ or Mg^2+^) in a conserved intramolecular coordination site consisting of aspartates, asparagine, and/or serine residues (D154, D156, D169, and N166 in the case of *M. villosus* ArlB1; Fig. 5E). Here, the coordinating metal ions have been implicated in increasing the stability of the archaellins [62, 64]. This does not appear to be the case for *O. meridianum* ArlB, where no metal ion was resolved. Indeed, the corresponding site does not contain a combination of amino acid residues that would be capable of coordinating ions (Fig. 5A).

### CdiP pilus machinery is distinct from that of Aap

We observed features in the CdiP machinery that distinguish it from previously characterized archaeal T4P, such as the twitching Aap pili of *S. acidocaldarius*. In general, the major and minor pilins AapB and AapA can either be encoded in the same gene cluster as the rest of the machinery or in a remote locus. In *H*.* volcanii,* it appears that even up to four distinct pilins, which are also located elsewhere in the genome, are assembled by the same ATPase and membrane platform (PilBC) complex [70]. Similarly, the *cdiPA* and *cdiPB* genes reside ~500 kbp away from the machinery operon, and 862 bp apart from each other (Fig. 3A). Our RNAseq data of cells grown to the mid-log phase and early stationary phase indicate that the pilins CdiPA and CdiPB are expressed at higher levels in the mid-log phase. However, there were no significant growth phase-dependent differences in the expression values of the remainder of the operon.

**Figure 6:**
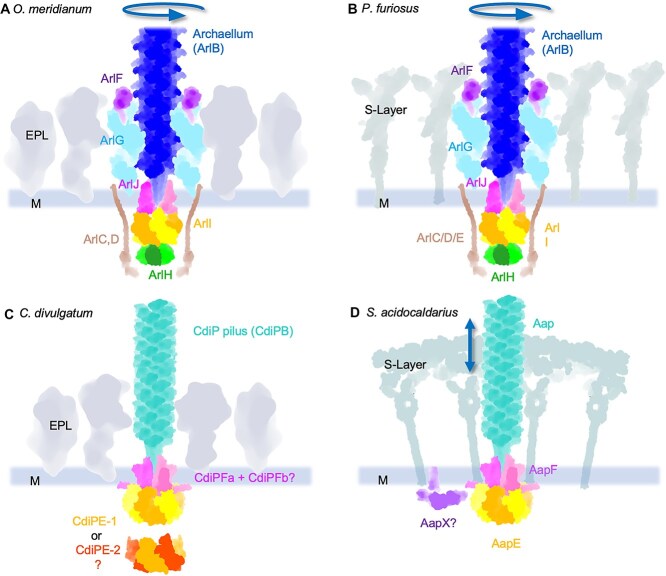
Working models of the *C. divulgatum* pilus and *O. meridianum* archaellum machinery, compared to homologues from *Pyrococcus furiosus* and *Sulfolobus acidocaldarius*. (A) The *O. meridianum* archaellum is typical for those found in *Methanobacteriati* (e.g. *P. *f*uriosus* (B). It consists of a homopolymeric filament composed of ArlB. The filament is assembled by the platform protein ArlJ, which is driven by the ATPase ArlI. Only a single ATPase is encoded in the archaellum operon, suggesting that ArlI provides the energy for filament assembly, as well as clockwise and anticlockwise rotations. ArlH has been proposed to be a molecular switch that shifts the archaellum machinery from assembly to rotation mode upon autophosphorylation. ArlC/D have been suggested to act as cytoplasmic stators that transmit chemotactic signals to the archaellum machinery. In species that are surrounded by an S-layer (B), ArlF/G are thought to form periplasmic filaments that anchor the archaellum machinery in the S-layer. Instead of an S-layer, *C. divulgatum* and *O. meridianum* are bounded by an unordered glycoprotein layer (EPL). The EPL may play an S-layer-like role in anchoring the archaellum machinery via ArlF/G. (C) The CdiP pilus machinery consists of a helical filament composed of the pilin CdiPB. A minor pilin, CdiPA, is encoded in the operon. Whereas its location is unknown, it may form a part of the pilus machinery. The filament is generated by a membrane-embedded platform protein. This platform is split into CdiPFa and CdiPFb, which together may assemble into a functional unit. The energy for the filament assembly is provided via ATP hydrolysis, catalysed by an AAA-ATPase. *C. divulgatum* encodes two such ATPases, CdiPE1 and CdiPE2. (D) Model of the Aap machinery, which functions in twitching motility in *S. *a*cidocaldarius.* In contrast to the CdiP machinery, the Aap machinery encodes only one ATPase (AapE) and one platform protein (AapF). The Aap operon also encodes AapX, the location and function of which is unknown. In A and C, the EPL is represented by random shapes and is not drawn to scale.

The CdiP machinery operon contains homologues of AapE (ATPase) and AapF (membrane platform protein), which we here refer to as CdiPE and CdiPF (Fig. 3A, Supplementary Fig. 6), respectively. In *C. divulgatum,* there are two copies of CdiPE (CdiPE1 and CdiPE2), and *cdiPF* is split into two independent genes (*cdiPFa* and *cdiPFb*; Figs 3A and 6A, Supplementary Figs 6 and 10). Moreover, *C. divulgatum* encodes no homologues for AapX in in the pilus operon. This is surprising, as AapX is an essential gene for generating Aap filaments in *Sulflobus* [20].

Multisequence alignments and Alphafold2 [44] predictions of CdiPFa and CdiPFb showed significant homology with the pilus platform protein AapF from *S. acidocaldarius*. Whereas CdiPFa aligns with the N-terminus of AapF, CdiPFb matches its C-terminal region (Supplementary Fig. 10). RNAseq indicates an uneven expression ratio of 2:1 for CdiPFa and CdiPFb (Fig. 3A). Although the functional significance of splitting the pilus platform protein into two differentially expressed halves remains to be clarified, it may point to a more modular assembly of the CdiP machinery compared to its counterpart in the Aap of *Sulfolobus* spp*.* (Fig. 6A).

Multisequence alignments and Alphafold2 [44], predictions of CdiPE1 and CdiPE2 in comparison with AapE from *S. acidocaldarius* indicate significant structure and sequence homology between the three ATPases (Supplementary Fig. 11). The finding of the two ATPases is unique, as archaeal operons encoding for pilus or archaellum machinery reported thus far all contain a single ATPase gene [20, 59, 71]. Bacterial T4P, however, often utilize two or more ATPases, especially those involved in twitching motility. Here, one family of ATPases is thought to power extension and the other pilus retraction [72, 73], and, at the first glance, this could indicate a similar role for CdiPE1 and CdiPE2 in *C. divulgatum*. Asking if *C. divulgatum* is indeed capable of twitching motility, we performed live cell microscopy. After image acquisition, we used a trainable detector [57], to measure the speed and displacement of cells. The cells mainly adhered to the surface of the substrate but did not display twitching motility (Supplementary Fig. 12, Movie 1). This is consistent with previous studies demonstrating adhesive properties of archaeal T4P and archaella [70, 74, 75].

### *O. meridianum* archaellum machinery

Following our finding that *O. meridianum* possesses an archaellum machinery akin to that of **Methano*bacteriota* (Fig. 6A and B), we performed RNAseq. This confirmed that all genes in the *O. meridianum* archaellum operon are expressed (Fig. 3B, D, F). In the early stationary phase, the expression is slightly elevated compared to the mid-log phase, and the relative differences in gene expression are consistent. Whereas the major archaellin gene *arlB* showed the highest expression levels, *arlF-J* have similar RNAseq count values. *arlC* and *arlD* show far higher transcription levels than *arlJ-F*, with *arlD* being comparable with that of the archaellin *arlB* (Fig. 3D). Such high expression levels suggest that ArlC and ArlD form assemblies with numerous subunits, such as within the proposed cytosolic rings [59].

Given the possibility that a lack of an S-layer is reflected in special adaptions in the subunits of the *O. meridianum* archaellum machinery*,* we compared it with that of *P. furiosus* via sequence alignments (Supplementary Fig. 13), and Alphafold2 [44], predictions (Supplementary Fig. 14). Together with sequence alignments. This analysis revealed that all machinery components are largely conserved on the structural level. The highest degree of conservation is observed between the homologues of ArlI, ArlJ, and ArlH, which form the core catalytic part of the archaellum machinery (Fig. 6A and B; Supplementary Fig. 14F–H). Despite the resemblance of the *O. meridianum* gene cluster with those of *Methanobacteriati,* our tomograms did not reveal a typical polar cap that is thought to coordinate archaellar bundles at one cell pole [59].

To test if the archaellum is functional, we subjected *O. meridianum* cells to swimming assays (Supplementary Fig. 15A, Supplementary Movies 2 and 3). As archaellation is regulated by growth conditions [76], we performed live cell imaging at different temperatures (40°C and at 55°C after 1 h of exposure to this temperature). We observed that *O. meridianum* is capable of directional swimming motion, thus confirming that the archaella of this organism are indeed functional. Motility was also seen in nutrient-depleted semi-solid gelrite plates at 40°C, whereas in plates at 45°C or with complete media, no swimming halo was observed (Supplementary Fig. 15B and C). Cells did not grow on plates at 55°C.

## Discussion

S-layers are paracrystalline protein coats commonly surrounding archaea [60, 77]. They provide key selection advantages, e.g. protecting the cells from viral attack or adverse extracellular conditions [5, 77, 78]. However, *Thermoplasmatales* mostly do not possess an S-layer, despite growing in extremely acidic environments. Our cryoET data confirm that *O. meridianum* and *C. divulgatum* lack a *bona fide* paracrystalline S-layer, in accordance with previous findings based on conventional electron microscopy [35], as well as cryoEM/ET of Ca. Scheffleriplasma hospitalis [29], and T*.* acidophilum [30]. However, close inspection of our tomograms revealed a dense, likely glycoprotein layer on the outer surface of the plasma membrane of both species. Although many similar densities were seen, this layer appeared overall heterogeneous and, in contrast to typical S-layers, unordered. S-layer proteins are usually amongst the most expressed in archaea; however interrogating our RNAseq data did not reveal the expression of any auspicious S-layer homologues. To uncover the identity of this protein layer, further studies, e.g. involving proteomics, are required. Nevertheless, the lack of a typical S-layer in these highly acidophilic species showcases that S-layers are not necessarily required as a protectant against extreme environmental pH values.

*Oxyplasma meridianum* produces an archaellum consisting of ArlB, which is encoded in an operon known from *Methanobacteriati*. Yet, a polar cap is missing, which suggests a level of divergence between *Methanobacteriati* and *Thermoplasmatales* that is not immediately apparent from the genetic background. The polar cap has been suggested to act as a polar anchoring structure for archaella, which apparently is not required in *O. meridianum.* In this context, it is notable that *Thermoplasmatales* were placed recently in a separate phylum; the *Thermoplasmatota* [26, 79].

The *O. meridianum* archaellum filament has an unusual, barbed structure distinct from previously investigated archaella, which tend to have a rather smooth surface [59, 62, 64, 66]. The barbs are formed by a unique extended loop that is flanked by two short ⍺-helices and spans from V126 to L153. On the genetic level, this loop is encoded by an insertion that is not present in other known archaella. Barbed filaments have previously been implicated in the formation of strong biofilms, e.g. the striking Hami filaments observed in *Candidatus* Altiarchaeon hamiconnexum [80]. Aside from swimming motility, archaella are equally important for adhesion. When archaeal cells are not engaged in swimming, the archaella are used for cell–cell and cell-surface attachment [21, 74, 81]. Thus, it is conceivable that the barbes of the *O. meridianum* archaellum also might aid in increasing the surface “grip” or the interlocking of archaella in biofilms [21, 81].

The *O. meridianum* archaellum displays an unusually high degree of glycosylation. Each ArlB is post-translationally modified with eight glycans, making it the most heavily N-glycosylated archaellin known to date. It has previously been proposed that N-glycosylation may confer resilience to extremely low pH values [82]. However, surveying the literature for a potential relationship between the number of N-glycans per subunit and the optimal growth temperature or pH did not show any correlation (Supplementary Tables 2 and 3). Some archaeal species have been shown to utilize O-glycosylation, e.g. the T4P of *Saccharolobus solfataricus* [65], *S. islandicus* [83], and threads of *S. acidocaldarius* [67]. Regardless, a link between O-glycosylation and the microorganism’s growth conditions is also not apparent. Thus, it seems that surface glycans have more subtle roles. For example, glycans are used as “recognition tags,” enabling *Sulfolobus* species to perform selective genetic exchange under ultraviolet (UV) stress within the same species but not with others [84]. Through interaction of UV pili with specific glycosylation sites on S-layers of the same species, genetic material is thought to be shared after species-specific aggregation [84]. Moreover, glycans have been shown to affect motility. For example, in *Halobacterium salinarium,* glycan truncation leads to increased bundling of archaella and compromised motile behaviour [85].

S-layer mutants in *S. islandicus* are incapable of swimming motility, although they do assemble archaellar filaments [86], suggesting that the S-layer is essential for this process. It has been proposed that the S-layer forms a scaffold for the archaellum machinery and acts as an anchor point for the stator subunits ArlG and ArlF [25]. ArlG is believed to form pseudoperiplasmic filaments [24] that surround the archaellum and reach up towards the S-layer [25]. The ArlG filaments are thought to be capped by ArlF, which, in turn, interacts with the S-layer [87]. As such, ArlG/F would act as stators that are essential to prevent futile rotation of the machinery in the membrane (Fig. 6D) and thus ensure proper swimming motility [25]. Extrapolating this hypothesis to *O. meridianum* now raises the question of how ArlG/ArlF can act as stators in the absence of an S-layer. Indeed, we find that *O. meridianum* shows swimming behaviour, like other S-layer-less archaea, such as *T*.* acidophilum* and *T. volcanium* [48, 88]. The answer may lie in the amorphous EPL, which, albeit being disordered, may still act as an anchoring matrix for the stators of the archaellum machinery (Fig. 6C).

In order to test whether *O. meridianum* ArlF shows structural differences that could account for a specialized motif evolved to bind irregular proteins distinct from S-layers, we compared Alphafold2 predictions [44] of ArlF from the S-layer-less *Thermoplasmatales* species *O. meridianum*, *T. acidophilum*,** and *T. volcanium* with those of S-layer-bounded archaea: *P. furiosus* [59], *S. acidocaldarius* [89], and *H*.* volcanii* [60], (Supplementary Figs 16 and 17). This analysis revealed minor differences between the ArlF homologues and thus no obviously distinct binding motif. However, *T. acidophilum* does not contain an ArlG homologue (Supplementary Figs 16 and 17), questioning the role of the putative periplasmic stators in archaellum function. To fully clarify the role of the S-layer, ArlG, and ArlF in the function of archaella, further experimentation, including high-resolution structures of archaellum motor complexes, will be required.

*Cuniculiplasma divulgatum* produces the CdiP filament, and its machinery components are similar to those of Aap from *Sulfolobales* [12]. However, various characteristics distinguish CdiP and Aap. In contrast to the twitching Aap of *S. acidocladarius* [12, 28]*,* the CdiP does not have the distinct three-conformer structure that has been proposed to aid the collapse of the Aap into the membrane during twitching motility [12], and indeed, we were unable to detect twitching motility in *C. divulgatum.* Another notable feature of the CdiPB subunit is its long hinge region. These hinge regions likely provide pili with flexibility [63], and it can be assumed that the flexibility of a pilus is proportional with the length of that hinge. It follows that the long hinge may have evolved to provide the CdiP filament with particularly high degrees of flexibility, which may aid surface adhesion, cell–cell contacts, and biofilm formation. In contrast, a typical hinge region for archaella consists of two residues [63], and the archaellum of *O. meridianum* follows this general trend. The shorter hinge likely confines the conformational space of archaellins, enabling them to form supercoils [63], which is an important prerequisite for swimming motility.

Whereas the genes encoding for the CdiP machinery appear to be related to those of T4P/Aap, the CdiP complex seemingly lacks an AapX homologues and the platform protein CdiPF is split into two distinct genes (*CdiPFa* and *CdiPFb*). Although it is believed that the AapF platform protein in T4P-generating *Sulfolobales* species forms a homodimer consisting of two copies of AapF, our data suggest that the *C. divulgatum* CdiPF platform could potentially form a modular oligomer, e.g. consisting of CdiPFa and CdiPFb in a ~2:1 stoichiometric ratio.

Operons of archaeal Aap pili, as well as all known archaella, encode a single multifunctional ATPase. In the Aap machinery, the ATPase AapE appears to power the assembly and retraction of the pilus. In the archaellum machinery, a single ATPase (ArlI) drives the assembly, as well as clockwise and anticlockwise rotation of the filament [12, 25, 28, 59, 69]. The *C. divulgatum* CdiP operon is an intriguing outlier, as it encodes two ATPases, CdiPE1 and CdiPE2. This is reminiscent of bacteria, which often encode two or more ATPases for T4P involved in twitching motility—one (PilB family), which drives pilus extension, and another one (PilT family), catalysing pilus retraction [90–92]. Considering this, the necessity for a second ATPase in the apparently immotile *C. divulgatum* therefore remains enigmatic.

## Supplementary Material

Supplementary-Figures-Final-6_wraf176

Movie1_Twitching_figureS13_wraf176

Movie2_Oxy_swimming_wraf176

Movie3_Oxy_swimming_wraf176

## Data Availability

The cryoEM raw data generated in this study have been deposited in the EMPIAR repository with the accession code EMPIAR-12897. The cryoEM maps have been deposited in the EM DataResource under accession codes EMD-19186 and EMD-19112 for *O. meridianum* and *C. divulgatum*, respectively. The corresponding atomic coordinates have been deposited in the Protein Data Bank database under accession codes PDB-8RH5 and PDB-8REY*.* The genomes of *O. meridianum* strain M1 and *C. divulgatum* strain S5 are available in NCBI GenBank under accession numbers CP133772.1 and NZ_LT671858, correspondingly. The RNAseq can be found in the form of spreadsheets in the supplementary data.
