# Supplementary material for: Unusual cell surfaces, pili, and archaella of Thermoplasmatales archaea
Source: ISME J. 2025 Aug 13;19(1):wraf176. doi: 10.1093/ismejo/wraf176 (PMC12448462; doi:10.1093/ismejo/wraf176)
Supplement: Supplementary-Figures-Final-6_wraf176 [file supplementary-figures-final-6_wraf176.pdf]

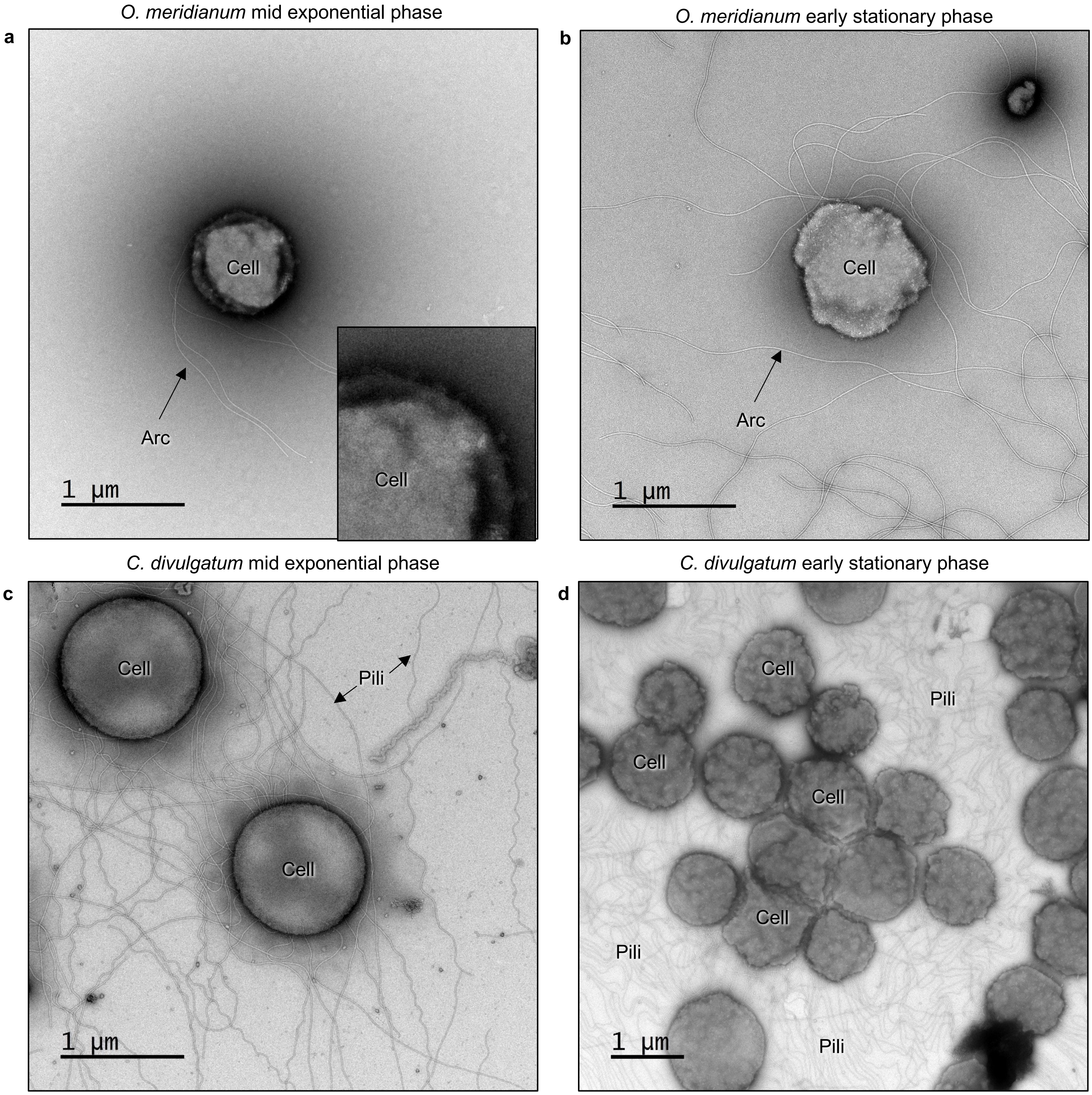

Supplementary Figure 1: *C. divulgatum* and *O. meridianum* change their morphology in early stationary phase. Negative stain micrographs of *O. meridianum* (**a**) and *C. divulgatum* (**c**) cells in mid exponential phase.

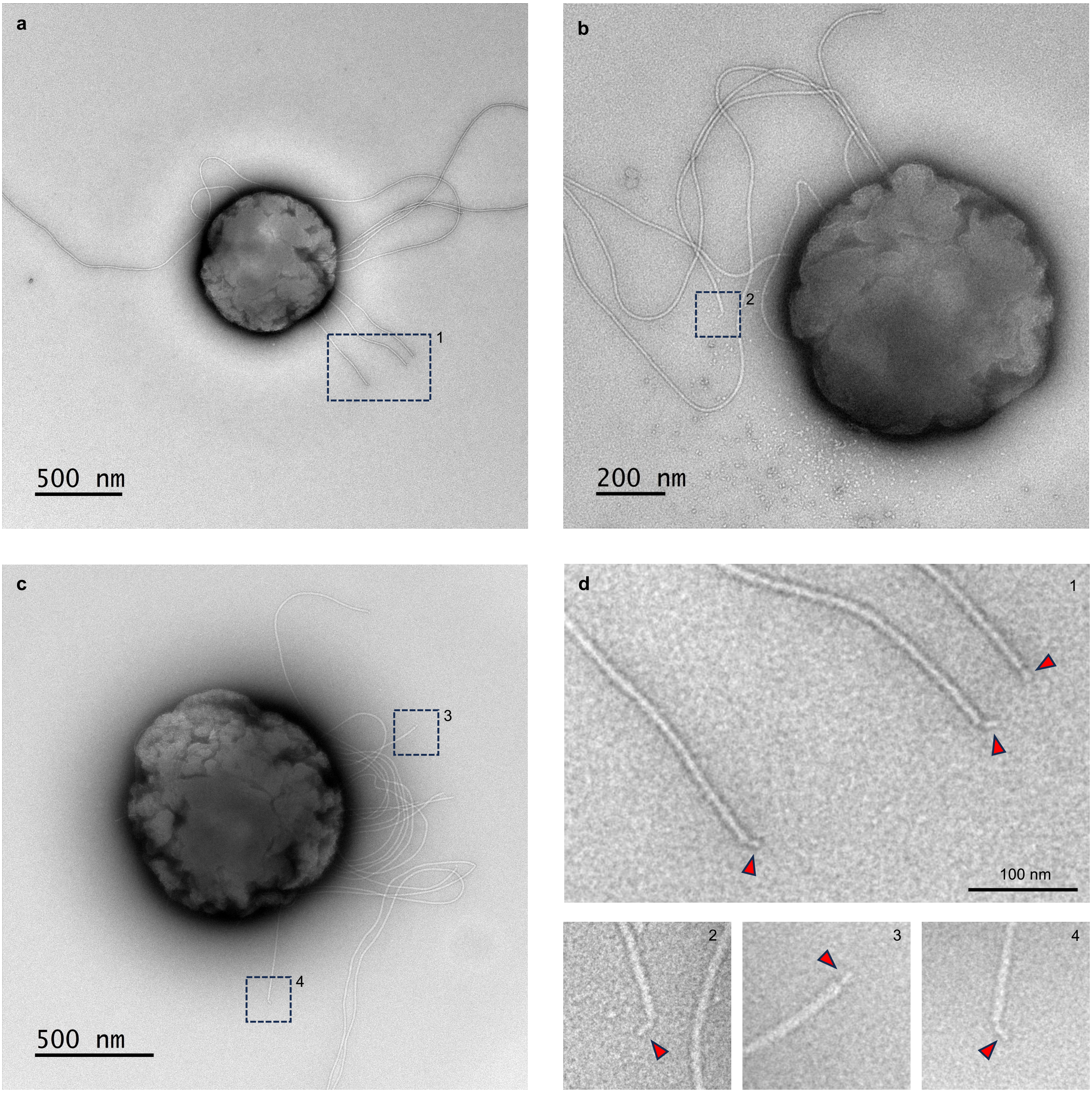

Supplementary Figure 2: ***C. divulgatum*** **filaments have hooked termini**
a-c, *C. divulgatum* cells extending pili-like filaments. **d**, close-ups of the distal termini of these filaments reveal hook-like densities. Close-ups numbered 1-4 in **d** relate to the numbered regions in **a-c**

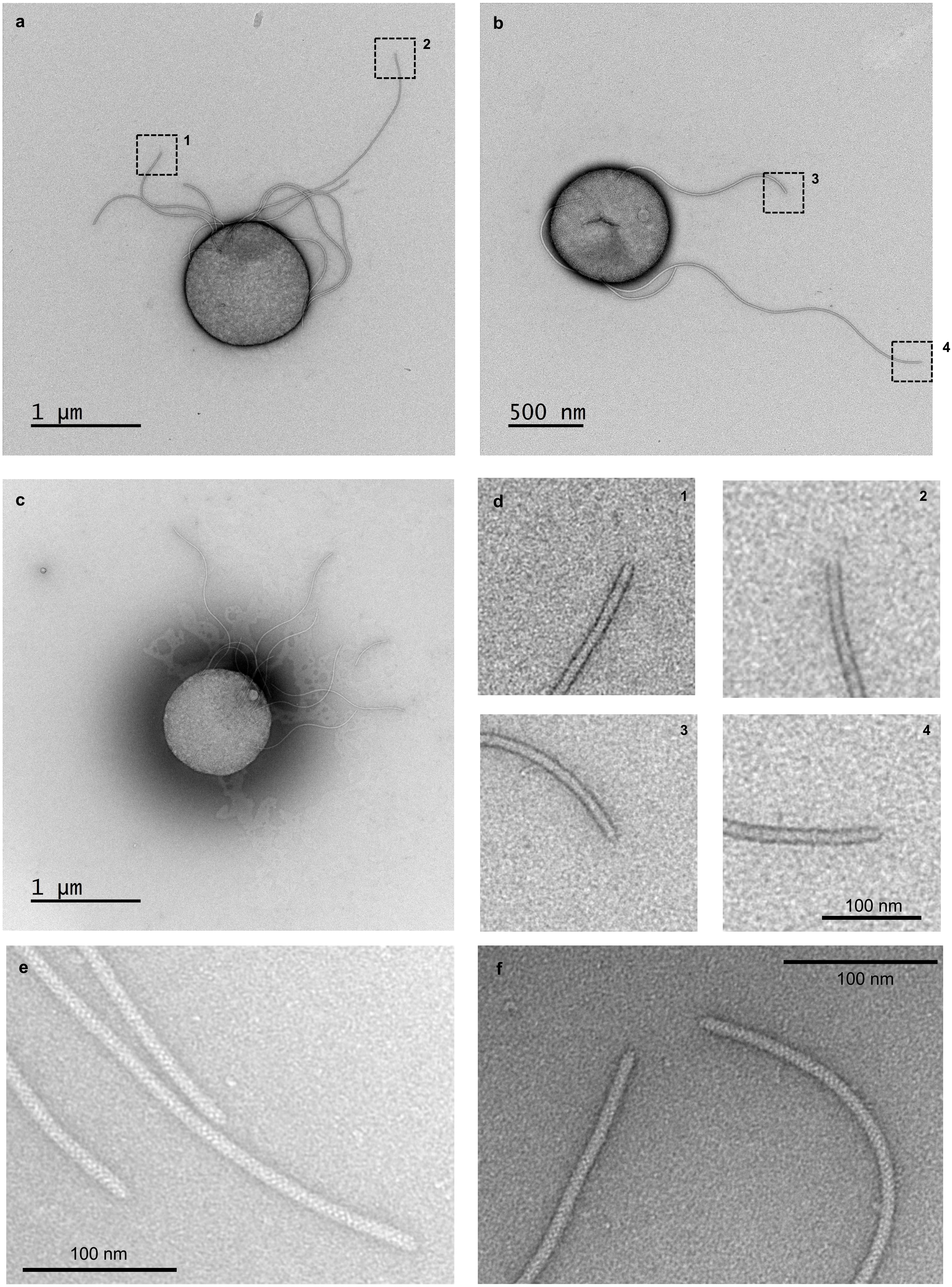

Supplementary Figure 3: *O. meridianum* filaments display blunt ends
a-c, *O. meridianum* cells extending archaella-like filaments. **d-f**, close-ups of the distal termini of these filaments do not show hooks but blunt ends instead. Closeups numbered 1-4 in **d** relate to the numbered regions in **a-c**

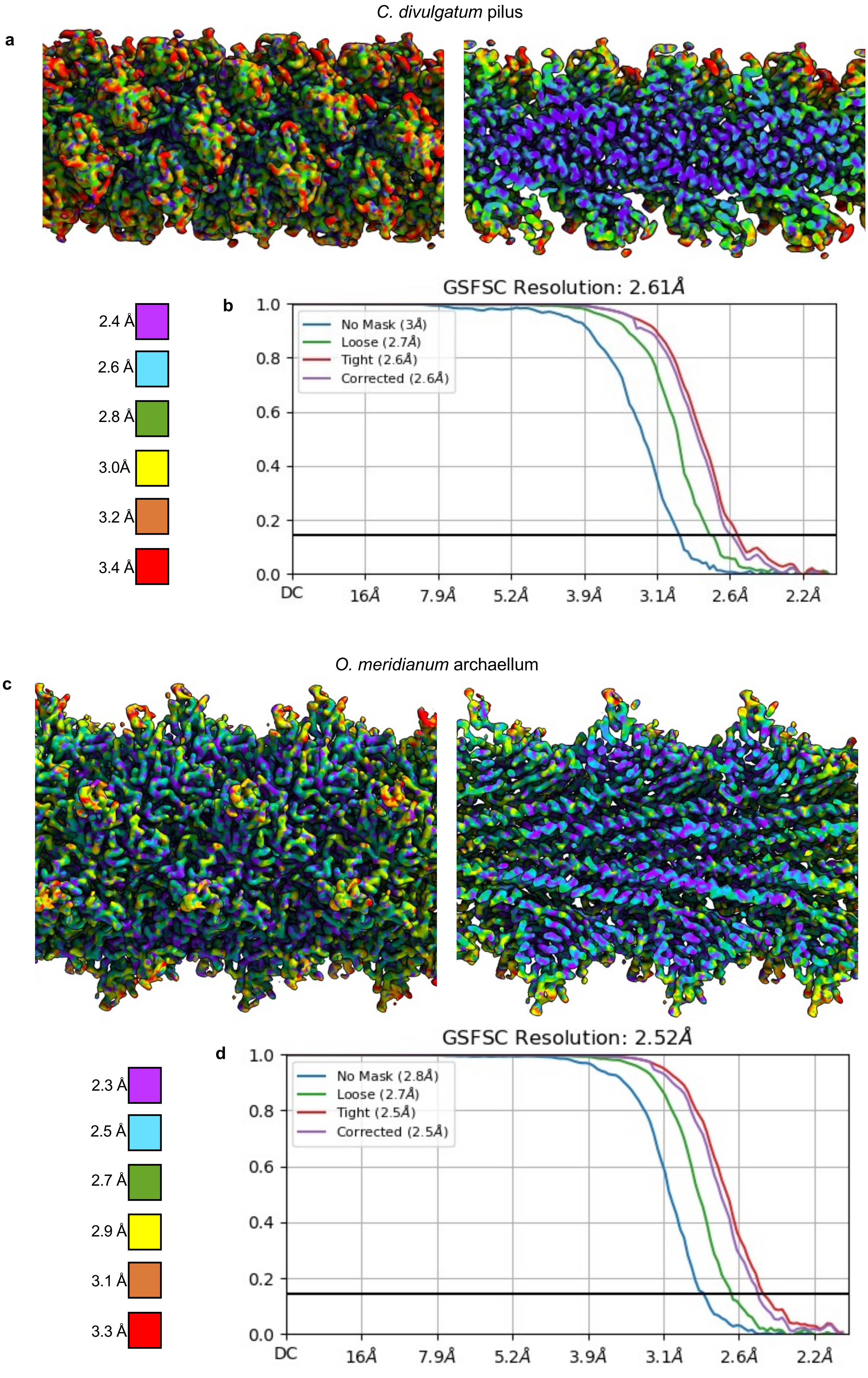

C. divulgatum pilus
a
2.4 Å
2.6 Å
2.8 Å
3.0Å
3.2 Å
3.4 Å
b
GSFSC Resolution: 2.61Å
No Mask (3Å)
Loose (2.7Å)
Tight (2.6Å)
Corrected (2.6Å)
1.0
0.8
0.6
0.4
0.2
0.0
DC
16Å
7.9Å
5.2Å
3.9Å
3.1Å
2.6Å
2.2Å
O. meridianum archaellum
c
2.3 Å
2.5 Å
2.7 Å
2.9 Å
3.1 Å
3.3 Å
d
GSFSC Resolution: 2.52Å
No Mask (2.8Å)
Loose (2.7Å)
Tight (2.5Å)
Corrected (2.5Å)
1.0
0.8
0.6
0.4
0.2
0.0
DC
16Å
7.9Å
5.2Å
3.9Å
3.1Å
2.6Å
2.2Å

Supplementary Figure 4: Resolution estimation

Local resolution maps of the *C. divulgatum* (**a**) and *O. meridianum* (**c**) filaments in surface view (left) and cross section (right). The global resolution was estimated using gold standard Fourier shell correlation (**b**, **d**).

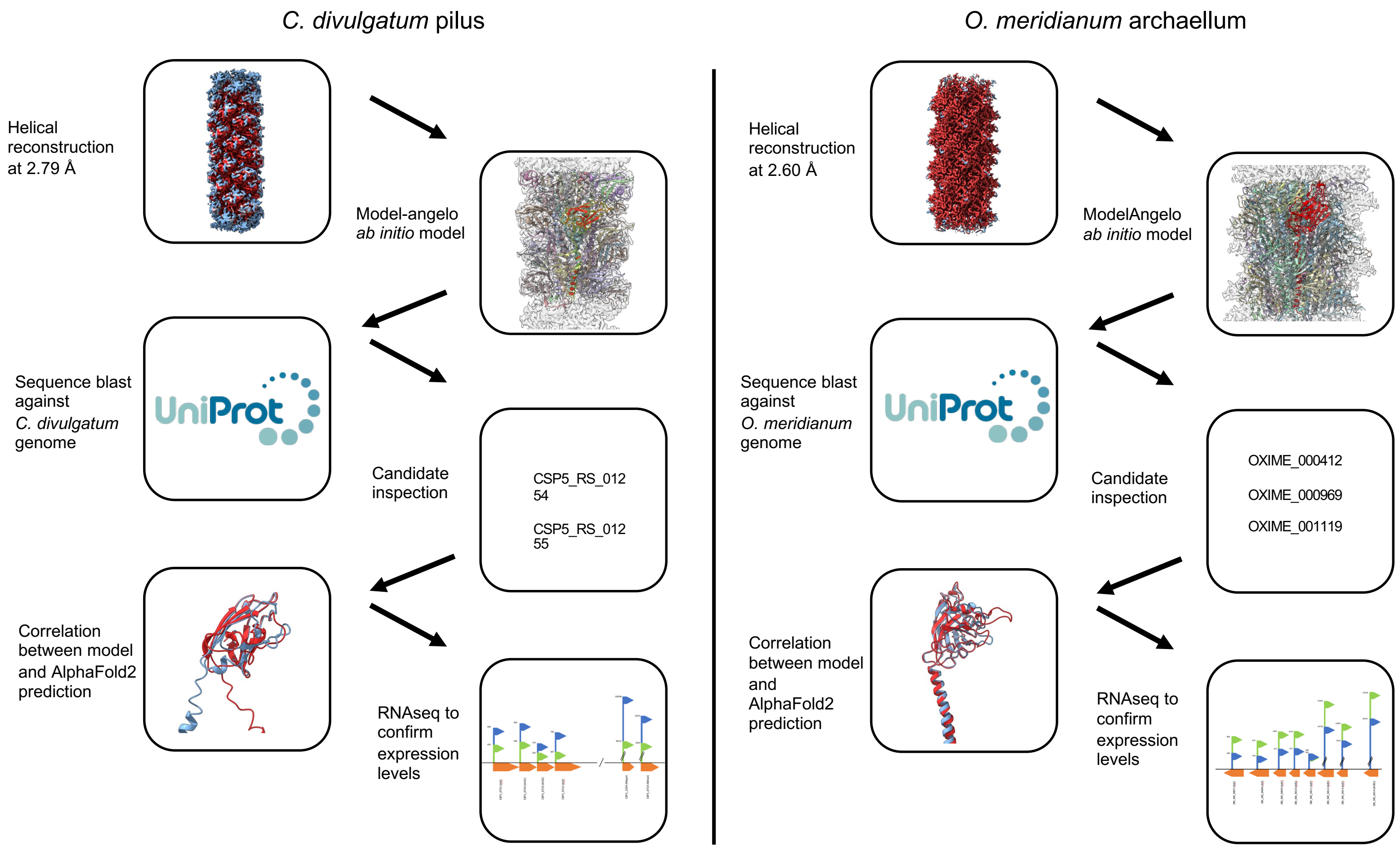

Supplementary Figure 5: “Visual proteomics” pipeline

Schematic diagram showing the pipeline for determining the major pilins/archaellins comprising the *C. divulgatum* and *O. meridianum* filaments. Following helical reconstruction, Model-Angelo [1], was implemented to determine an initial amino acid sequence for the major pilin proteins. Sequence blast against both genomes was performed. The potential candidate sequences were subjected to Alphafold2 [2], and the resulting structural predictions compared against an ab-initio structure from the map. The expression of the gene candidates was then confirmed via the mRNAseq data.

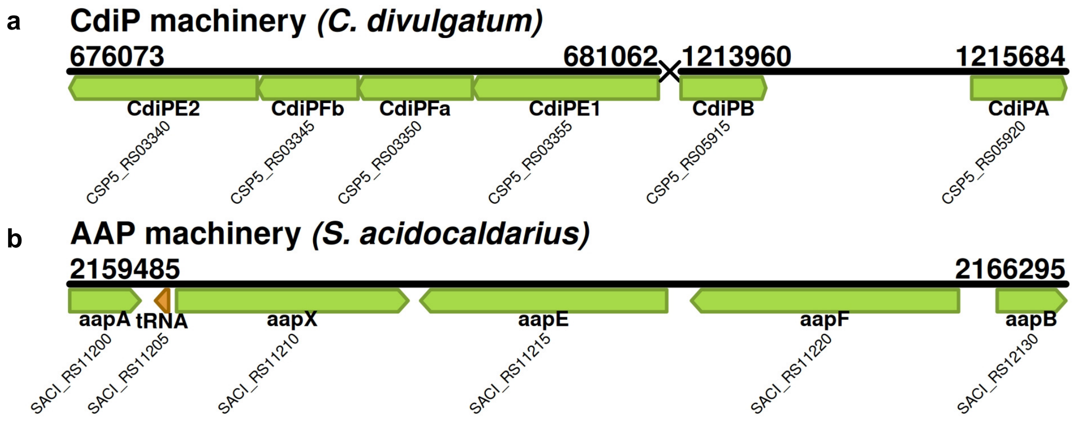

Supplementary Figure 6: The operon of the CdiP pilus machinery of *C. divulgatum.*
The operon of the CdiP pilus machinery of *C. divulgatum* (**a**) compared with that of the Aap machinery of *Sulfolobus acidocaldarius* [3] (**b**).

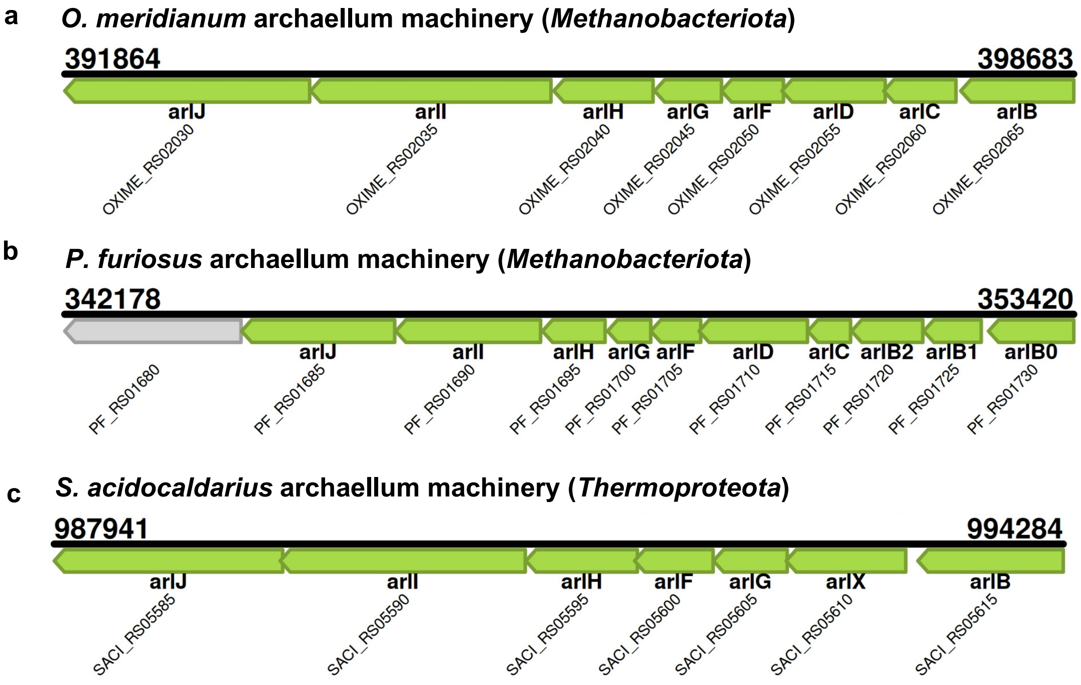

Supplementary Figure 7: The predicted operon of the *O. meridianum* archaellum machinery. a, the *O. meridianum* archaellum operon compared with archaellum operons of the *Pyrococcus furious* (*Methanobacteriota*) [4] (**b**) and *Sulfolobus acidocaldarius* [3] belonging to the phylum (*Thermoproteota*) (**c**).

a *C. divulgatum* **pilin (CSP5_RS05915; CdiPB)**

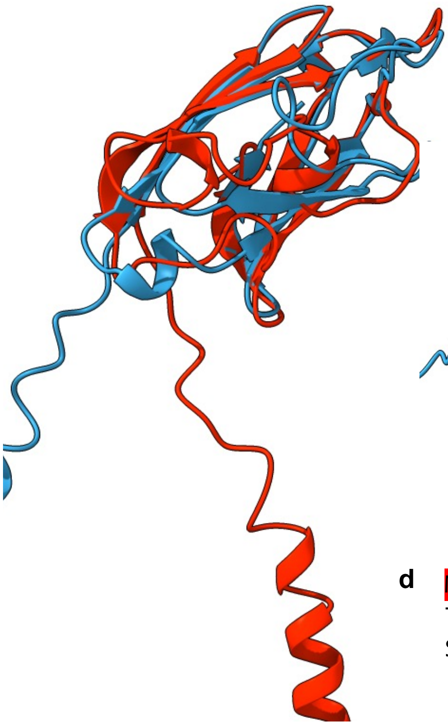

b

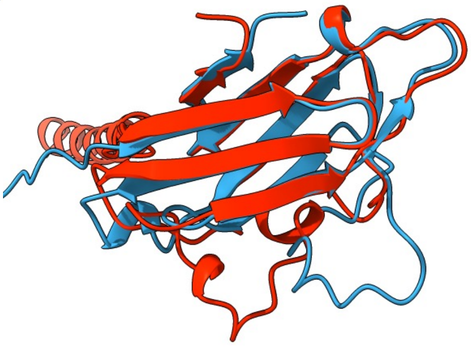

c

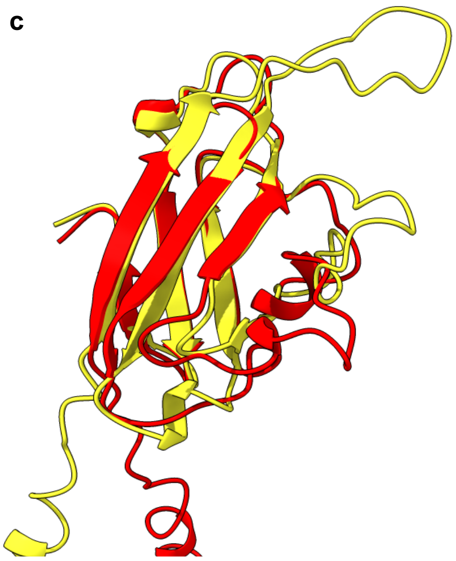

d

MKELRPKEDKAVSPIIATILLIAITVVLAATLVTILGGFTHGVSNTVETAGV
TSHITSKYIFINVSSSSSAISASSITITITGASFKVTSGDTLAEVAGVSSTS
SNATFTGGSDYTVPISLSSSQTVAGVSFELIYKGNVIYNSAA

e *O. meridianum* **archaellin (OXI_ME_00412; ArlB)**

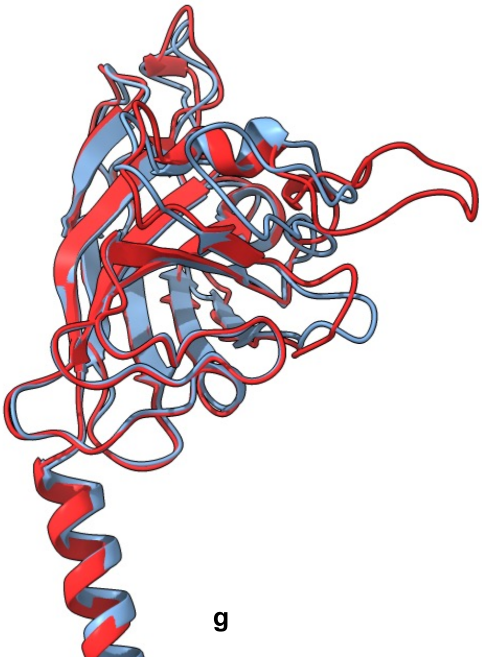

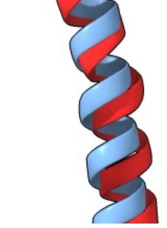

f

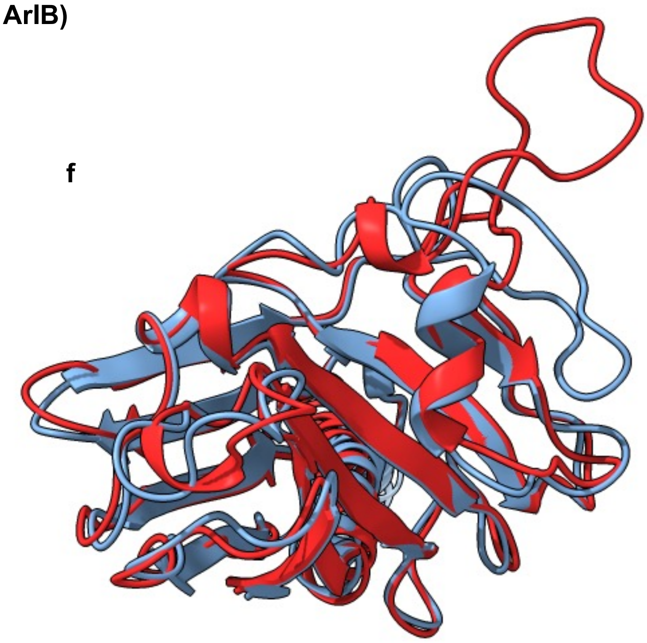

g

MYRAVYRGVKKLRKVFSLKADNKAETGIGTLIIFIAMVLVAAVAATVLINTAGSLQQRAT
STGSQTTNQVSTGLIVQSIYGMDNNRSNPESGSLNWTAIYVTLNTGSSPVDLSNVSLSLE
YQGQLASLKYTPATTNASFAVDTNGTSNVFSVLNAGVGYKNSTATFKNVELKNVTKSTNF
AIVVIRDPSNSLTSSHPVLTTGSEVVILVNTSAVFGGMKQGQAVTGQINPSVGSPGIIQF
TTPSAFTETVMELQ

Supplementary Figure 8: Experimental structures vs. Alphafold2 predictions
a, b, superimposition of the Alphafold2 [2] prediction of *C. divulgatum* CdiPB (blue) and the structure determined from the CryoEM map (red) in side view **(a)** and top view **(b)**. **c,** comparison between the solved CdiPB structure (red) and the Alphafold2 [2] prediction of CdiPA (yellow). **d,** amino acid sequence of CdiPB, showing the cleaved signal sequence in red, and glycosylation sites in yellow. **e, f,** superimposition of the Alphafold2 [2] prediction of *O. meridianum* ArlB (blue) and the determined structure (red) in side view **(e)** and top view **(f)**. **g,** amino acid sequence of *O. meridianum* ArlB, showing the cleaved signal sequence in red, and glycosylation sites in yellow.

a

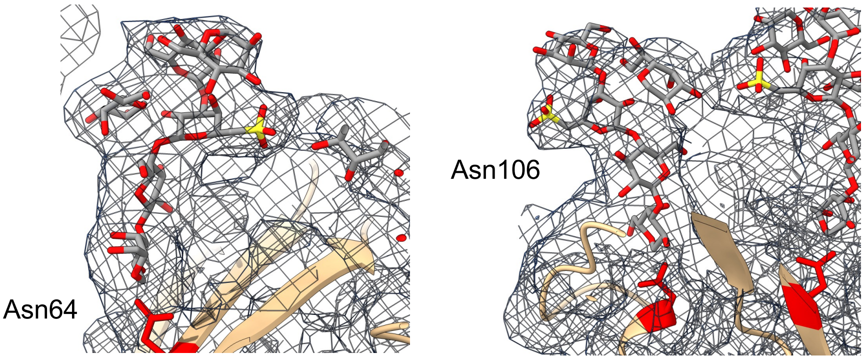

b

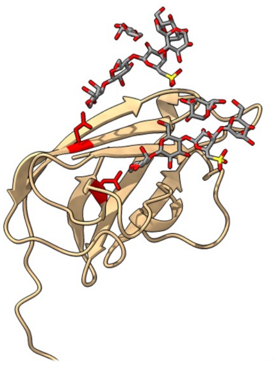

c

***C. divulgatum* CdiP**
VSPIIATILLIAITVVLAATLVTILGGFTHGVSNTVETAGVTSHITSKYIFINVSSSSSAISASSITITITGASFKVTSGDTLAEVAGVSSTSSNATF
TGGSDYTVPISLSSSQTVAGVSFELIYKGNVIYNSAA

d

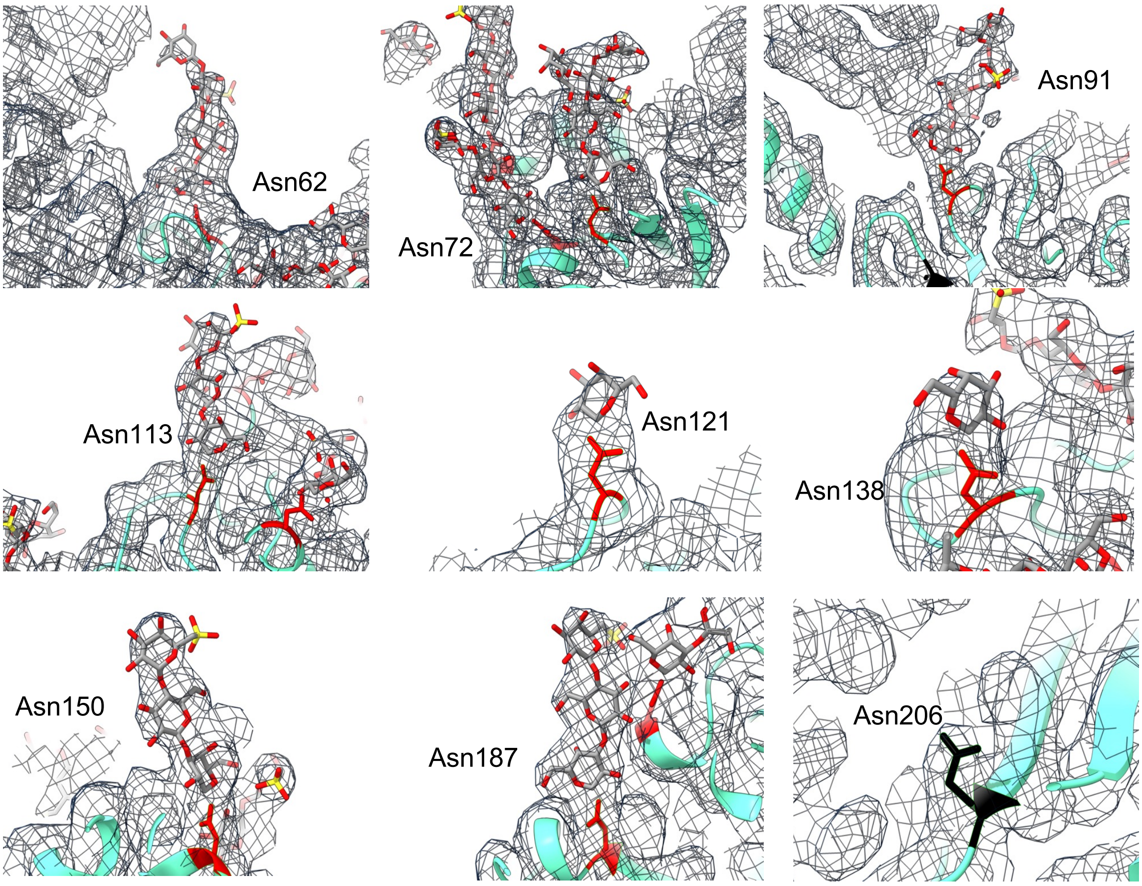

e

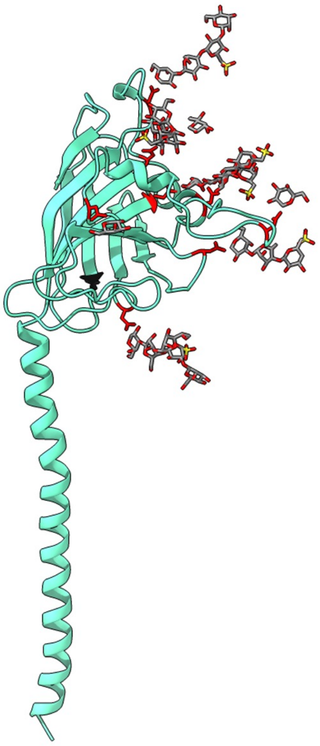

f

***O. meridianum* ArlB**
ETGIGTLIIFIAMVLVAAVAATVLINTAGSLQQRATSTGSQTTNQVSTGLIVQSIYGMDNNRSNPESGSLNWTAIYVTLNTGSSPVDLSNVSLSLEYQGQ
LASLKYTPATTNASFAVDTNGTSNVFSVLNAGVGYKNSTATFKNVELKNVTKSTNFAIVVIRDPSNSLTSSHPVLTTGSEVVILVNTSAVFGGMKQGQAV
TGQINPSVGSPGIIQFTTPSAFTETVMELQ

Supplementary Figure 9: Glycosylation sites in the *C. divulgatum* pilin and the *O. meridianum* archaellin. a, close-ups of the gycosylation sites in the *C. divulgatum* pilin. The cryoEM map is shown as grey mesh, the protein backbone as beige ribbon, and glycans as sticks colored by element. The glycosylated residues Asn64 and Asn 106 are labelled red. **b,** Structure of the head of the *C. divulgatum* pilin (beige ribbon). The glycans are shown as sticks. **c,** amino acid sequence of CdiPB. Glycosylation sites are labelled yellow. **d,** close-ups of the gycosylation sites in the *O. meridianum* archaellin. The cryoEM map is shown as grey mesh, protein backbone as light blue ribbon, and glycans as sticks colored by element. The 8 glycosylated residues Asn62, Asn72, Asn91, Asn113, Asn121, Asn138, Asn150, Asn187 are labelled red. One N-glycosylation sequon remains unglycosylated (Asn206) **e,** Structure of the head of the *O. meridianum* archaellin (light blue ribbon), the glycans are shown as sticks. **f,** amino acid sequence of the *O. meridianum* archaellin ArlB. Glycosylation sites are labelled yellow.

a

```
C. div. CdiPFa,b    MHKSQDIQIKKENKSNGFLSNLNKKLNPKPVKVVIPAPTIFDVFAVRVFGHLVEKYVQTE    60
S. aci. AapF        ------------------MSRMSK---DKKSSSNVNIPSIYLLFYHTPVVKRLAGY----    35
                                      :*.:.*    *   .   :   *:*: :*    . : :   *

C. div. CdiPFa,b    KMDESLRKAKMPIDAIEYYSRGIMIAVIFMV--VSFIAVNI------F-SLKFPSYTYLS    111
S. aci. AapF        -FDKRLLTSRNPEDPKLFASRLFLILLVCIVLAVMFISFALIIFLRFYRVTLLPAYLALS    94
                     :*: * .:: * *    : ** ::* :: :*  * **:. :       :    :*:*  **

C. div. CdiPFa,b    FAFWFLAVIV----YFAVMAGYPNSIAGTRRKKIDAVLPLAMGYIATMASADMPVENIMY    167
S. aci. AapF        LVMLFLGVIIPPIAYLISILDISQKIDKIKNGVDAESFSFATLFVIFLKSGLSPV-LLFR    153
                    :.: **.**:    *:  : .   :.*   :.      : :*   ::  : *.   **  ::

C. div. CdiPFa,b    ELSNSTEYGELAREAKSIAVSTRLFGEDIINAVKDGAANSPSQRLSEFFQGIITTLTSGG    227
S. aci. AapF        KLEGSKAFSFVQDIVVYVNRRVQYLSESIEQALLKAMDINPGKLFNDFMLAYVTAIRTGA    213
                    :*..*. :. :    .   :    .: :.*.* :*: ..    .*.: :.:*: . :*:: :*.

C. div. CdiPFa,b    DLKSYFKDKAVQYQTELSTLIKRNTESLSVLAESYIIV---GIMFPLILMVIIGTVTSVI    284
S. aci. AapF        PVIETMEAKLKDLSKQFSLAANLASDRLQGVAESYVVWLSSGYI-MLYLVLIMGAILPFV    272
                    : . :: *   : ..::*    :   :: *. :****::     * :   * *::*:*::   .:

C. div. CdiPFa,b    PGEGSLTDTVLYLIVFLIIPIIAVMFALILSSTIGEVDVMKGEIGGVELRYVALAGSVLT    344
S. aci. AapF        G-NSSSLLTVLGPVVVLVVPMVNLLFVYMADSLQL--KFPETQSSAYKIFYISLPIGLVV    329
                    :.*    ***   :*.*::*:: ::*. : .*      .. : : .. :: *::*   .::.

C. div. CdiPFa,b    -MFVLFFT---GYFFKINDLNPLVHPLTLSSIAMVILFFPFGLADFARVKRIEE---AER    397
S. aci. AapF        AFLIMIFEHQIVYFITLSGGLQNVFPVSIALLI--GLLIASAPPAFFYQKEMREKSGFEE    387
                    :::::*      **:.:..     *.*:::: :     *::   .    *    *.:.*     *.

C. div. CdiPFabb    RLPDFLRDLAGHTNFGTPMSEAILR-SAENKYEPLSIEIEHLAGMVKLGIPVETALNDFG    456
S. aci. AapF        YAVKFLNAISEGLLAGLTFESIVTRLKDAQEMGKFREVLRKVDGYLKLGYPLTIALKRGA    447
                     .**. ::       *   :.. : * .   :: * :    :.:: * :*** *:   **:   .

C. div. CdiPFa,b    KRLKSP----SIIRVGKIIKKASESGSNTSDVISLV-SSFTTQTYLMRESRFADMRSYST    511
S. aci. AapF        DSINEFTSRIALYTLSDM----IEIGSMTPDNVRALADQINSQL-VVRREYQGKVK---P    499
                    . ::.      ::  :..:      * ** * * :   : ..:.:*   ::*..   ..::

C. div. CdiPFa,b    TLATSFGVFLFVIVMLDTFFFPQIAGGGLSGGGVLNL---ASSSYGLIEK--------LF    560
S. aci. AapF        LIATPYAGVL--VSLIATFLLA---------SGILSMLNSGIAVYGPIATGLVSIPQIIF    548
                    :** :. .*   : :: **::            .*:*.:    . : ** * .         :*

C. div. CdiPFa,b    --SAGVIVQSAASGLISGVFRDGRLTSGAMMSGILVLISIMVLAIIGVL-----------    607
S. aci. AapF        ITAISGILNAFLAGLLIGKIGYGKASAGF-IHGIILMIVV-TLTIFAFVELRISIVPNFH    606
                    : . *:::   :**: * :    *: ::*   : **:::* : .*:*:..:

C. div. CdiPFa,b    -----                                                           607
S. aci. AapF        SNISF                                                           611
```

b

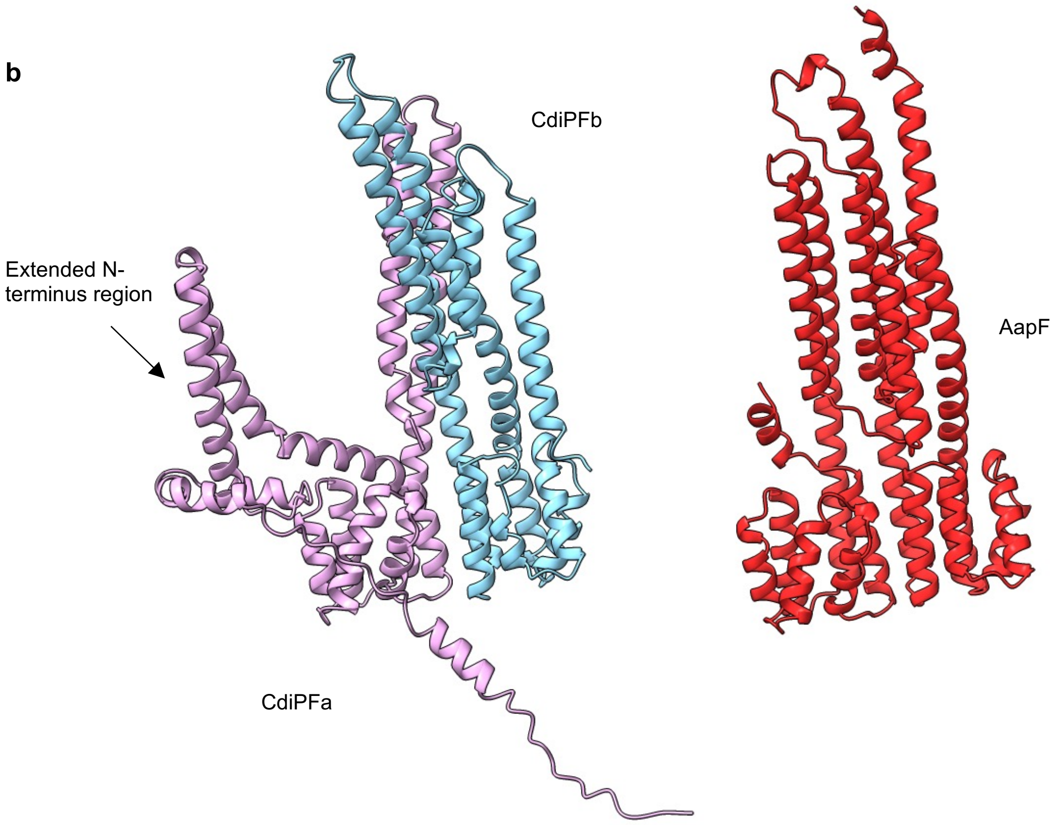

Supplementary Figure 10: Comparison between the membrane platform homologs CdiPFa/b found in *C. divulgatum* and AapF from *S.acidocaldarius.* a, sequence alignment between *C. divulgatum* CdiPFa (magenta) combined with *C. divulgatum* CdiPFa CdiPFb (cyan) and AapF from *S. acidocaldarius*. **b,** Alphafold2 [2] predictions of CdiPFa (pink) joined to CdiPFb (blue) and AapF (red)**.**

a

```
C. div. CdiPE1    MPL--FSPTKKKEK------------KPVKNVPSKREERITEVPSVGKYEILDEYNIIPQ 46
S.aci. AapE       MALSKFTRKSNKDLEEVSQYKIPATLYPLTGIPEEV----LNIS--SEYE-VDILSQIPK 53
                  * *  *: ..:*:             *:..:*.:     ::   .:** :*  . **:

C. div. CdiPE1    TVTV----------------EISWNPTSMESIYIANEPVLSSDEEILLKKISKNMEQ-IV 89
S.aci. AapE       ELVDQLNNSGIEMKIPNPHVFIYFDNDKGVHKYVLVEPPLEEKSFVIYKLLIKYLERQFV 113
                   :.                 * ::  .    *:  ** *.... :: * : * :*: :*

C. div. CdiPE1    KSSKNLP---EDLSQLTIEKVIDSYLKGRKYKLNRRTVDKIKYFIKRNYEGYEPLEPVVR 146
S.aci. AapE       GKHFDIGASIRDFSNIYRDYVIEG—SRGDYTVLKPEARVALYHIIRNLLGYNVFTPLLA 171
                   .  ::    .*:*::  : **:.  .  .*.: :  .    *.* **  **. : *::

C. div. CdiPE1    DPLVEDVSCNGVGIPVYVEHKIHGSLKTNIVFDNE-----------KDLDSYVVRLAQMC 195
S.aci. AapE       DYKVEDISVNGLNVPIYVYHRDYEYIPTNIIFQKRMSVLNEEVDGEEFLNQITMRYISLS 231
                  *  ***:* **:.:*:** *: :  : ***:*::.           : *:. .:*  .:.

C. div. CdiPE1    GKEVSMNEPIIDGTFPQGHRIQLTFGNEISTKGSAFTFRLFRESPFTPVELIKYGAATSE 255
S.aci. AapE       GRSISIASPISDGMLPAGDRIAATYGREVSAKGTSFVIRRFSESPITVLHLIKQGVISAD 291
                  *:.:*: .** ** :* *.**  *:*.*:*:**::*.:* * ***:* :.*** *. :::

C. div. CdiPE1    LATYLWFAVENLKSAIIAGVPGVGKTSTINAILMFIPSNTKVFSIEETREINIMHQNWVA 315
S.aci. AapE       LVAYLWYAIDLKMSFMVIGTTGAGKTTVLNALLNLVKDTMKIVSIEDIPELKLARDNWVQ 351
                  *.:***:*::   * :: *. *.***:.:**:* :: .. *:.***:  *::: ::***

C. div. CdiPE1    TSTRESIYSTANGDSKNPPIGMFELVKMAMRQRPTYIVLGEIRGRESYSLFQAISTGHTA 375
S.aci. AapE       LYTRPVF------GALGKEITLMDLLKLSLRYRPDIITVGEIRGEESYVLFQALSTGHGG 405
                    **  :      .: .  * :::*:*:::* **  *.:*****.*** ****:**** .

C. div. CdiPE1    YSTIHADSMETLVNRLESNPLNIPRVLIGALNIVIFNKFVRVGSRNMRKLMEIDEIVGTD 435
S.aci. AapE       ATTFHAYDPESAIKRLMNEPLNIPRDWIPMMNVVLTVRRIPFGLSFKRRVVEVDEVVSYN 465
                   :*:** . *: ::** .:******  *  :*:*:  : : .*    *:::*:**:*. :

C. div. CdiPE1    QDSGEIMYNKVFSYDFNQNEQVFSGFS-KILNEIRDAKQWTQEEMNFEFERRKSLLDGLV 494
S.aci. AapE       KLK------KVGSWDPNNDSHTFDLSKSEVLKSRIEEIGLNLDIVGDEIKRRANYLKMLS 519
                  : .      ** *:* *::.:.*.  . ::*:.  :    . : :. *::** . *. *

C. div. CdiPE1    -----KNNITDYTNITRIINIYYKDPETAMKMASGI-----                   525
S.aci. AapE       DIKQILDRPDAYVFVKKYIVLYSIKPDEAIKEVQRMSVAKV                   560
                        :.   *. :.: * :*  .*: *:* .. :
```

b

```
C. div. CdiPE2    MAL--FSKTKKKKT-KKRKFLSRQVITP-----------------------VRTVKRTN 33
S.aci. AapE       MALSKFTRKSNKDLEEVSQYKIPATLYPLTGIPEEVLNISSEYEVDILSQIPKELVDQLN 60
                  ***  *::...:*.  :  ::    .: *                           . *.: *

C. div. CdiPE2    EDKFFQELIPGTVFSRVYFDRGATIFRYEVNEPEVKERVAKLYAKYRN----EFIASITD 89
S.aci. AapE       NSGI-EMKIP-NPHVFIYFDNDKGVHKYVLVEPPLEEKSFVIYKLLIKYLERQFVGKHFD 118
                  :. : :  ** . .  :***..  :..* : ** ::*: :   :*    :    :*:..  *

C. div. CdiPE2    --PEMYNISGLTKDDAVDQFTKALRMTNSFTPDEMDSLTYYVERDIKGFGKIDPIMRDAN 147
S.aci. AapE       IGASIRDFSNIYRDYVIE----GSRGDYTVLKPEARVALYHIIRNLLGYNVFTPLLADYK 174
                     .: ::*.: :* .::    . *   :.   *     *:: *:: *:. : *:: * :

C. div. CdiPE2    IEDISCDAPGVPVFVYHKAFGFIPTNIIYDDVEE-----------LETFVKKILQDVGRH 196
S.aci. AapE       VEDISVNGLNVPIYVYHRDYEYIPTNIIFQKRMSVLNEEVDGEEFLNQITMRYISLSGRS 234
                  :**** :. .**::***: : :******::.  .           *: :. : :.  **

C. div. CdiPE2    ISISNPIVDATLEDGSRISASIGKYITNKGSSFTIRKFKEEPLTPLDLIQRSLCNTRVFA 256
S.aci. AapE       ISIASPISDGMLPAGDRIAATYGREVSAKGTSFVIRRFSESPITVLHLIKQGVISADLVA 294
                  ***:.** *. *  *.**:*: *: :: **:**.**:*.*.*:* *.**::..: .: :.*

C. div. CdiPE2    YFWLLTEYGGNVMVVGGTATGKTTLMNAILLFIPAQKKIVSIEDTREINLVHENWVPMVT 316
S.aci. AapE       YLWYAIDLKMSFMVIGTTGAGKTTVLNALLNLVKDTMKIVSIEDIPELKLARDNWVQLYT 354
                  *:*   :   ..**:* *.:****::**:* ::    *******  *::*.::*** : *

C. div. CdiPE2    RTGYGKLDPETGKRAGEIDMFDLLTVTMRQRPSYVIVGEVRGAEAFTLFQAMSIGRYVYG 376
S.aci. AapE       RPVFGALG-------KEITLMDLLKLSLRYRPDIITVGEIRGEESYVLFQALSTGHGGAT 407
                  *  :* *.       ** ::***.:::* **. : ***:** *::.****:* *:

C. div. CdiPE2    TFHADDISTFIHRMESKPINIPRNLLLTLDVAVILQNVTDDRGSRRRIKTISEIAGQDSI 436
S.aci. AapE       TFHAYDPESAIKRLMNEPLNIPRDWIPMMNVVLTVRRIPFGLSFKRRVVEVDEVVSYNKL 467
                  **** * .: *:*: .:*:****: :  ::*.: ::.:  . . :**:  :.*:.. :.:

C. div. CdiPE2    SKDILINNAFILNEQSDTQNYSGFSYAIKKIAEKEGKFEDELEKEIDLRTRILDKMIE-K 495
S.aci. AapE       KKV----GSWDPNNDSHTFDLSKSE-VLKSRIEEIGLNLDIVGDEIKRRANYLKMLSDIK 522
                  .*      .::  *::*.* : *  . .:*.  *: *   * : .**. *:. *. : : *

C. div. CdiPE2    GISNYKDFYYMVNQFYKDRRRVFQSLDMTEEIQV---------                 529
S.aci. AapE       QILDRPDAYVFVKKYI-----VLYSIKPDEAIKEVQRMSVAKV                 560
                   * :  * * :*::      *: *:.  * *:
```

c

```
C. div. CdiPE1    MPLFSPTKKKEKKPVKNVPSKREERITEVPSVGKYEILDEYNIIPQTVTVEISWNPTSME 60
C. div. CdiPE2    MALFSKTKKKKTKKRKFLSRQVITPVRTVKRTNEDKFF--QELIPGTVFSRVYFDRGATI 58
                  * *** ****:.*  * :  :    :  *  ..: :::   ::** **  .: ::  :

C. div. CdiPE1    SIYIANEPVLSSDEEILLKKISKNMEQIVKSSKNLPEDLSQLTIEKVIDSYLKGRK--YK 118
C. div. CdiPE2    FRYEVNEPEVKERVAKLYAKYRNEFI--ASITDPEMYNISGLTKDDAVDQFTKALRMTNS 116
                    * .*** :..    *  *  :::   .. :.    ::* ** :..:*.: *. :   .

C. div. CdiPE1    LNRRTVDKIKYFIKRNYEGYEPLEPVVRDPLVEDVSCNGVGIPVYVEHKIHGSLKTNIVF 178
C. div. CdiPE2    FTPDEMDSLTYYVERDIKGFGKIDPIMRDANIEDISCDAPGVPVFVYHKAFGFIPTNIIY 176
                  :.   :*.:.*:::*: :*:* ::*::**  :**:**:. *:**:* ** .* : ***::

C. div. CdiPE1    DNEKDLDSYVVRLAQMCGKEVSMNEPIIDGTFPQGHRIQLTFGNEISTKGSAFTFRLFRE 238
C. div. CdiPE2    DDVEELETFVKKILQDVGRHISISNPIVDATLEDGSRISASIGKYITNKGSSFTIRKFKE 236
                  *: ::*:::* :: *  *:..:*..:**:*.*: :* **. ::*: *:.***:**:* *:*

C. div. CdiPE1    SPFTPVELIKYGAATSELATYLWFAVENLKSAIIAGVPGVGKTSTINAILMFIPSNTKVF 298
C. div. CdiPE2    EPLTPLDLIQRSLCNTRVFAYFWLLTEYGGNVMVVGGTATGKTTLMNAILLFIPAQKKIV 296
                  .*:**::**: . ..:.: :*:*: .*   ..::.*  ..***: :****:***::.*:.

C. div. CdiPE1    SIEETREINIMHQNWVATSTRESIYSTA-NGDSKNPPIGMFELVKMAMRQRPTYIVLGEI 357
C. div. CdiPE2    SIEDTREINLVHENWVPMVTRTGYGKLDPETGKRAGEIDMFDLLTVTMRQRPSYVIVGEV 356
                  ***:*****::*:***   ** .  .   : ..:   *.**:*:..:*****:*:::**:

C. div. CdiPE1    RGRESYSLFQAISTGHTAYSTIHADSMETLVNRLESNPLNIPRVLIGALNIVIFNKFVRV 417
C. div. CdiPE2    RGAEAFTLFQAMSIGRYVYGTFHADDISTFIHRMESKPINIPRNLLLTLDVAVILQNVTD 416
                  ** *:::****:* *: .*.*:***.:.*:::*:**:*:**** *: :*::.:: : *

C. div. CdiPE1    GSRNMRKLMEIDEIVGTDQDSGEIMYNKVFSYDFNQNEQVFSGFSKILNEIRDAKQWTQE 477
C. div. CdiPE2    DRGSRRRIKTISEIAGQDSISKDILINNAFILNEQSDTQNYSGFSYAIKKIAEKEGKFED 476
                  .  . *::  *.**.* *. * :*: *:.*  : :.: * :****  :::* : :   ::

C. div. CdiPE1    EMNFEFERRKSLLDGLVKNNITDYTNITRIINIYYKDPETAMKMAS---GI--    525
C. div. CdiPE2    ELEKEIDLRTRILDKMIEKGISNYKDFYYMVNQFYKDRRRVFQSLDMTEEIQV    529
                  *:: *:: *. :** ::::.*::*.::  ::* :*** . .::  .    *
```

d

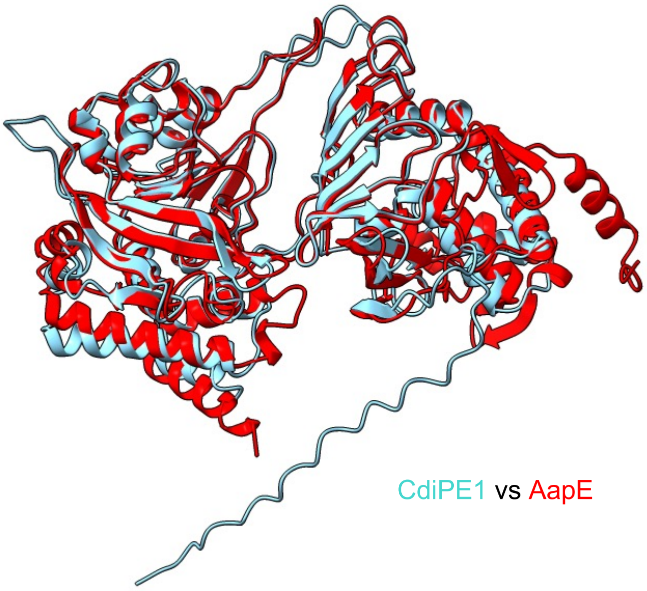

e

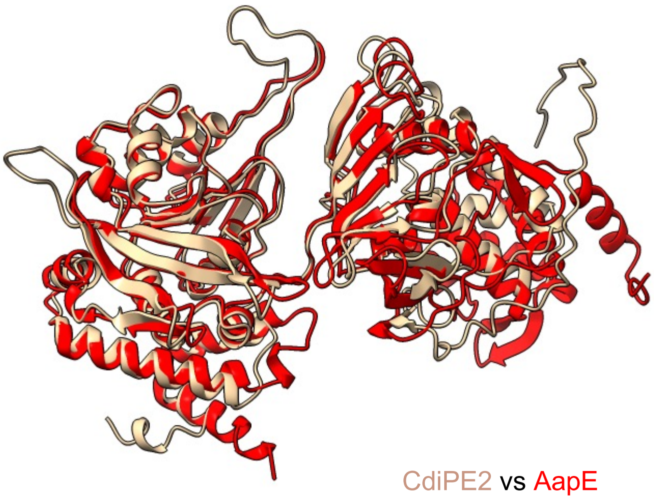

f

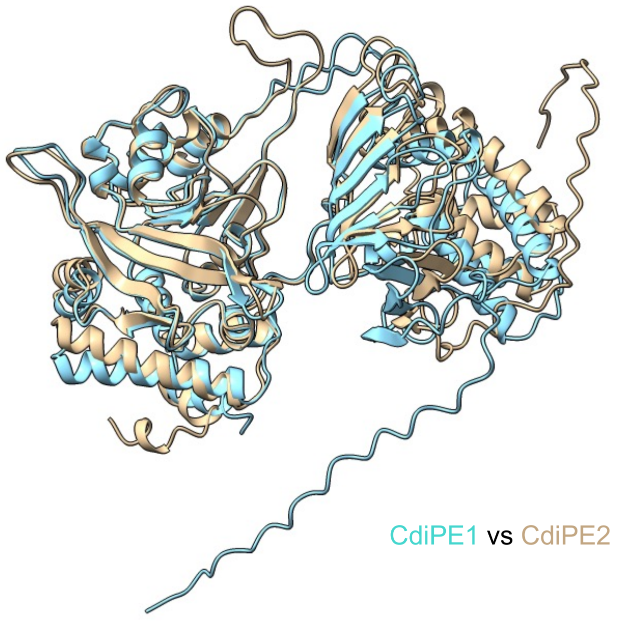

Supplementary Figure 11: Comparison between the ATPases CdiPE1 and CdiPE1 of *C. divulgatum* with AapE of *S. acidocaldarius.* Amino acid sequence alignment between *C. divulgatum* CdiPE1 and *S. acidocaldarius* AapE **(a)**, *C. divulgatum* CdiPE2 and *S. acidocaldarius* AapE **(b),** and *C. divulgatum* CdiPE1 with CdiPE2 **(c)**. Superimposition of the Alphafold2 [2] predictions of *C. divulgatum* CdiPE1 (blue) and *S. acidocaldarius* AapE (red) **(d);** *C. divulgatum* CdiPE2 (brown) and *S. acidocaldarius* AapE (red) **(e);** *C. divulgatum* CdiPE1 (blue) and *C. divulgatum* CdiPE2 (brown) **(f).**

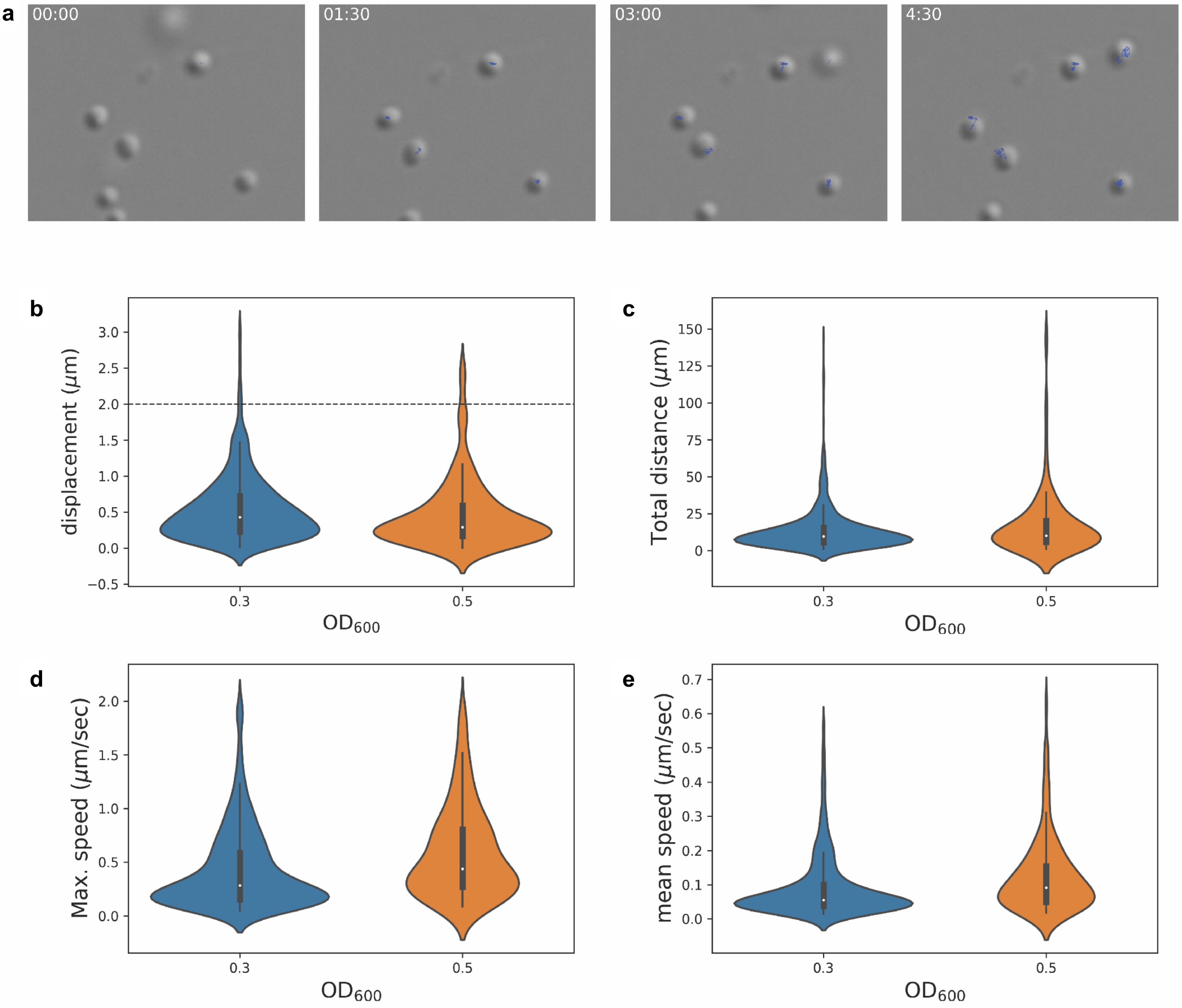

Supplementary Figure 12: *C. divulgatum* does not display twitching motility
a, live cell microscopy of C*. divulgatum* cells at 40°C and OD_{600} of 0.3. DIC microscopy images showing different time points during the total observation window. Tracks are shown in blue. **b, c**, total and maximum distances traveled by the cells. **d, e**, maximum and mean speed of cells. The observation window was of 5 minutes in every case.

```
O. meri. ArlB     MYRAVYRGVKKLRKVFSLKADNKAETGIGTLIIFIAMVLVAAVAATVLINTAGSLQQRAT     60
P. fu. ArlB0      ------------------MAKKGAVGIGTLIVFIAMVLVAAVAAAVLIQTSGYLQQKSQ     41
                                     :*. .******:***********:***:*:* ***::

O. meri. ArlB     STGSQTTNQVSTGLIVQSIYGMDNNRSNPESGSLNWTAIYVTLNTGSSPVDLSNVSLSLE     120
P. fu. ArlB0      ATGRETTQEVASGIKVLSVVGKTDSN----KTYVEKLAIYISPNAGSEGIDLNNTRVVLS     97
                  :** :**::*::*: * *: *  :..    .  ::  ***:: *:**. :**.*. : *.

O. meri. ArlB     YQGQLASLKYTPATTNASFAVDTNGTSNVFSVLNAGVGYKNSTATFKNVELKNVTKSTNF     180
P. fu. ArlB0      NGTVQAVLKYEKTAYHKGA---------VGDVFNAS-------------TAWNLSNTNF     134
                       * ***  :: : .          * .*:**.                    ..***

O. meri. ArlB     AIVVIRDPSNSLTSSHPVLTTGSEVVILVNTSAV---F-GGMKQGQAVTGQINPSVGSPG     236
P. fu. ArlB0      GIIVLQDADNSVDQNYPTLNKGDIVVITVKVGEGNGVFGKGIPPRTKITGKVIPEFGAPG     194
                  .*:*::* .**: ..:*.*..*. *** *:..     *  *:     :**:: *..*:**

O. meri. ArlB     IIQFTTPSAFTETVMELQ  254
P. fu. ArlB0      VIEFTTPSTYTSEVIELQ  212
                  :*:*****::*. *:***
```

```
O. meri.ArlC      --MGLLDDIFGRRRRETT-GNVATQNVPQQQPPGPNQGAPSQFDSKFIDGVNQRITSTEN     57
P. fu. ArlC       MPLDFLSSLFKRKKEEEVEGEQEEINVEELEER-----VENREENEQLNQIMERINEIEN     55
                    :.:*..:* *::.* . *:    ** : :        . .: :.: :: : :**.. **

O. meri. ArlC     EISKINNALDGAKRSISDIRTELDVIKENVKMVVSLYEMVSKKFNPFMDTTPEEIKEI--     115
P. fu. ArlC       DIPRIKISIDNIKKQIQELREEIERLDKTIKDVMMLYEVVSQEINPFKEQLSQETSLSSE     115
                  :* :*: ::*. *:.*.::* *:: :.:.:* *: ***:**:::*** :   :* .

O. meri. ArlC     TDALREEIHEVELLVRSAINDLRELYGT-PDVDGLVSEIEPEVDEDDQ                 162
P. fu.ArlC        IQELKKQIEEIRMELAQVKNDIKVLAGYGVDIDSILYEVLAEV-----                 158
                   : *:::*.*:.: : .. **:: * *   *:*.:: *:  **
```

```
O. meri. ArlF     ------------MGFSYVAAVAILLSSSLIF---FGIIYSDYVHSETQLSNAQNDQNRQM     45
P. fu. ArlF       MSSMESEVGGIPIGVQRFSK-----CSQIIFISFLGAAITLYAAWDSAYADVKSA-QEFW     54
                              :*.. .:      .*.:**   :*   : *.  ::  ::.:.  :.

O. meri.ArlF      YDYINSKVK-ITGYDVSPY-----NSVYNVTINMTNTGSISLDLMNSNL-------LLNG     92
P. fu. ArlF       YDLKISQMSTDFTYNETPGHVYFSPTTYSITLVLINTGRTLYAPYWSGVYDGRYITLYNV     114
                  **   *::.    *: :*        :.*.:*: : ***         *.:       * *

O. meri.ArlF      SLENFSYSA--EYLLPMANGSVS-----FQTTAGNYQVEIA---------FNTGYNV---     133
P. fu. ArlF       SDDNVGFSENYTYILPGDYFQITIANIPRDTTTHNLTITVENGCWFRLSWYYDGTTVLVL     174
                  * :*..:*    *:**    .::      :**: *  : :           :  * .*

O. meri.ArlF      ------ITEVKV                                                     139
P.fu. ArlF        PIQRGCPTEVD-                                                     185
                        ***.
```

```
O. meri. ArlG     -MASIATSEMIMFIATMVLTASVVGVLGGETLHLSNSLTDSSASASTQIQTDFKIINDPS     59
P. fu. ArlG       MAAGEPASELILFIVAVIVASTVAGALAYVTTDIANGMKDRGKLLANQLRVEFAIINDPQ     60
                    *.  :**:*:**.:::::::*.*.*.  * .::*.:.* .   :.*::.:* *****.

O. meri. ArlG     QIPK----DKGYVFYVKNTGQVGFDFTNNTVSTIINGSIVTGGYISYITPAGSN-GELMP     114
P. fu. ArlaG      SVRVTGTGPYTYTFYIKNIGKETIPFTSSSVQVFIDGNLIPPSNLTFKDVNGNQITSLKP     120
                  .:          *.**:** *:  : **..:*..:*:*.::  . :::    *.:  .* *

O. meri. ArlG     SQVGTIVVNV---TLSPGYHTIVITLNDGVTQQMVFEID-                         150
P. fu. ArlG       YEVGVLEVTLGNPLDTTTTHRIVVVLENGKKRSLVFRVKS                         160
                       :**.: *.:     :   * **:.*::* .:.:**.:.
```

```
O. meri. ArlI    MAEMIISDSLEVAQGRNPHLKAYLDRYRANTQRTPTFIPVPDNSLRDLASIDIIYPVGDP     60
P. fu. ArlI      -MAEVMSQTLEDAMRRNPHLRKYVEEFRRKYGKIPEFHVQLSRDMKEIPYPNIIYPVGDP     59
                     ::*::** *  *****: *::.:* :  : * *    ...::::   :********

O. meri. ArlI    IFIHINLDEFGEKTYTPIEPNLTDEEDHFM-DEVLNLILAVAADYPPPENDEVLESNLDE     119
P. fu. ArlI      IFIHIYGDPNTEKQYIVIEPRIETKEEEEKYKLIRDRMLELAPNKDIPEEQEEFERFLDS     119
                  *****  *   ** *  ***.:  :*:.   . : : :* :* :   **::* :*  **.

O. meri. ArlI     IYKKKVSLGTANKIKGTSATVTPELNSRLLYNLKKNVMGLGPIEPLIMDSNIEDISGIGA     179
P. fu. ArlI       LFEEAVLSL-VKGR--KGITITKEEMEKFRYLIKRDIIGIEPLEPIARDPYIEDIHIIGA     176
                  :::: *    .:    .. *:* *  .:: * :*::::*:**:**:  *  ****  ***

O. meri. ArlI     FDIFIVHKIFGTLRTRITFGNNDKLSLYSKRLSERVGRPVSDKTPIVDATLPDGSRLSIV     239
P. fu. ArlI       HYVSLVHKIFEHLPTNIKWKDNVELADYFKNLSERIGRPVSDRNPIVDGSLPDGSRINII     236
                  . : :*****  * *.*.: :* :*: * *.****:******:.****.:******:.*:

O. meri. ArlI     YSSDVSARGPSFTIRKFADIPISMVQLVKYNTLSAEEAAYLWLALEYGQSIFVCGETASG     299
P. fu. ArlI       YSPDISLKGPSATIRKFAATPLSITQLVAWGTMSAEIAAYLWLAIEYGMSIFVCGETASG     296
                  ** *:* :*** ******  *:*:.*** :.*:*** *******:*** ***********

O. meri. ArlI     KTTVINAMIPFIRPESKIFSAEDTPEIRAPQPAWQQLVTRERGAEDSRVTLYDLLRTALR     359
P. fu. ArlI       KTTTLNAIIPFIKPGSKVYTAEDTPEVQVPHPTWQRLVTRERGPEESRVTLFDLLKAALR     356
                  ***.:**:****:* **::******::.*:*:**:******* *:*****:***::***

O. meri. ArlI     SRPNYIIVGEIRGAEGAMAFQAMQTGHPVLSTFHASSVKKMIQRFTGTPINIPVTFMDNL     419
P. fu. ArlI       SRPNYIIVGEIRGAEGAIAFQAMQTGHPVMSTFHAGDVKKMIQRFTGSPINVPITFIDNL     416
                  ****************:**********:*****..**********:***:*:**:***

O. meri. ArlI     NIALIQQAVYVNGRFLRRTTSINEIEGYFEELGGVSTKQVFQWDPTTDQHVFRGLNNSYI     479
P. fu. ArlI       NIAIFQQAVYVKGKFLRRVINVVEIEGYYEELGGVATRNVFEWDPVSDKHIFRGMNNSYI     476
                  ***::******:*:****. .: *****:******:*::**:***.:*:*:***:*****

O. meri. ArlI     LEKRIAQTAGMRDPKAIYDELFKRTRIIEGLIRKNVNSYYDVFNITKTFYIKGEGAISAL     539
P. fu. ArlI       LEKKIAEIAGYEDPKDIYDELFLRARIIEKMVELKIFNYWDVYREIKAFYQRGLEGLSFR     536
                  ***:**: ** .*** ****** *:**** ::. :: .*:**:.  *:** :*  .:*

O. meri. ArlI     YG                                                               541
P. fu. ArlaI      I-                                                               537
```

```
O. meri. ArlD     ------------------------------------------------------------     0
P. fu. ArlD       MEELGYVTDAEINAKLAELKGKVPSVVINELREKLMAKKDSLTPRQLDEIVKRVLEAYGS     60

O. meri. ArlD     ------------------------------------------------------------     0
P. fu. ArlD       QAAKYEQISKRVDELGKKLSDLSSQLARLVEALEEKKFAVHEKKAEAVAEKAAEVSEKIE     120

O. meri. ArlD     ----MISE-SLKGYLQSKLDSADKSIP-------------KFVIEELRQKVLNS-----     36
P. fu. ArlD       KIEEMLEEKPRKSEIEKKLEELHRKIEELSERVAKEKVEETKARIEELEEKIAKGEEIKP     180
                      *:.*   *. ::.**:. .:.*              *  ****.:*: :.

O. meri. ArlD     ------------PTKFSKSDIETLFNNLQQR--------YSEGKENSLLGKVDTLFKEIT     76
P. fu. ArlD       EEIKEIEEEVEELGEFEEAPVEEIVVEEEKPPEPVEEVPVVEEKKEEVIEEV--PVEEVV     238
                               :*.:: :* :. : ::           * *::.:: :*   .:*:.

O. meri. ArlD     NVERMLSGDTNRKKPAEEGEGEDQQ------SLRNRNVWRTHRKSKLEAIGDDVISLMVA     130
P. fu. ArlD       EEEKVPSHVVEEEKVEEEGGREMAEKMKIPEDIASLLFEEEPRKARLEEIPDDVVSIMIA     298
                  : *:: *  .:.:*  ***  *  :      .: .  . .  **::** * ***:*:*:*

O. meri. ArlD     LRWLEFLLDNYGPENTIDILDYYESIGWISSNVKEQMIKFTKMTGIITPVQEY--KIKTT     188
P. fu. ArlD       LKWLGFLIDRVGIQNLEKVLEFYYDIGWISEKVFNQLLRFAKGTRPHHRDPEWKPAEKLT     358
                  *:** **:*. * :*  .:*::* .*****.:* :*::::*:* *       *:    * *

O. meri. ArlD     IQDHIVTMLFIEKLNGGEISRDIVESLEREFRTLKKGVDELYGI                     232
P. fu. ArlD       VQDHLISLLFIERLRGLRITRDVLDKLEREIKMLEKTLDEFYGV                     402
                  :***::::****:*.* .*:**:::.****:: *:* :**:**:
```

```
O. meri. ArlH     MFLNFTIEGDELDRRMGGGLYVGQIVTIIGGNGEGKTLLSLRLSYGVMKHGTEIAYISTQ     60
P. fu. ArlH       ------------------------MLIEGDRGTGKSIISQRLLYGFLMNGYTASYVSSQ     35
                                          : * *..* **:::* ** **.: :*   :*:*:*

O. meri. ArlH     FPIREFVSEAEALGYSLFNPILSGQVSYITTVFLLRK--SRRSTVDEMLNNEGIKEKQMV     118
P. fu. ArlH       YTTVEYVKQMVSIGYDIMPFLIRKKLVFVSLYPLLSGVSEERRFLSRLLGEPRLWEPDVV     95
                  :   *:*.:  ::**.::  ::  :: :::   **    ..*  :...:*.:  : * ::*

O. meri. ArlH     LIDSFKKDIFRDFNLSD---FFSKL-RKFSEGRVVILTVNPQDFTNEELTMIKQLSTTII     174
P. fu. ArlH       IIDSFSSVLSREEELKSVRNFLMYLKRLSSLGKVIILTANPDEIPRDTLFLLEEASTLLM     155
                  :****.. : *: :*..   *:  * *  * *:*:***.**::: .: * :::: ** ::

O. meri. ArlH     NLTSKDLAGERKHIIDLMKYPMAMKSFQQAIPFRIEPGRGLIVEISSVS                223
P. fu. ArlH       RLNVRVFGGDLKNSATIVKYNNAKGVFQKIIPFRVEPKVGLVVEIAAVV                204
                  .*. : :.*: *:   ::**  *   **: ****:**  **:***::*
```

```
O. meri. ArlJ     MNDAKRLGRFSGIEFGKKEVIFVLFAAAISGVFLMLYG-----E----TKN-IVFIIIAA     50
P. fu. ArlJ       MPE-EKASIFVKADIDPKEYMKKILIPGIVGSFATLLAIMIFTRIFTLPTGLKVFMYL--     57
                  * : :: . *   ::. ** :  ::  .* * *  * .     .     ..  **: :

O. meri. ArlJ     ASAVMLMAIFLKPVLERDNRKNNINSNIPFFITAFATLSVSGANRIDIIELLSKKDKLGF     110
P. fu. ArlJ       IPIILMFYTTAYPYLIADSKKIAINSKIPYFITYFAVLSTSEIGRSDLLKVMAQDPKLGA     117
                     ::::     * *  *.:*  ***:**:*** **.**.*  .* *:::::::. ***

O. meri. ArlJ     LKDDMVKLVNLVKNWKRGLSEAAMFLSQRTPSEVFADFLARFGHAIDSGQDFEEFVRDEA     170
P. fu. ArlJ       IAKELKKVYVLVDKFHRSLPEAFRFLARRTPSRVFADFLDRLAYSLDSGVELKEYLFQEQ     177
                  : .:: *:  **.:::*.* **  **::****.****** *:.:::*** :::*:: :*

O. meri. ArlJ     GTVMGNFETTYISSLYTFDLYKDMYVSLLLAFAFLITFILIMPILIPINIIAVLSLSMIT     230
P. fu. ArlJ       QTVMDDYQTFYEGALYDLDIFKEIYESIIISVVFAGAFIIIEPLITGQDIGRLALYLIFM     237
                   ***.:::* * .:** :*::*::* *:::::..*  :**:* *::   :*  :    ::

O. meri. ArlJ     IIMGEGLLVYGIKIVLPNDPIWHDTGIKTELQIKIRRIFIMAGVLSMV-LLTALLATGLY     289
P. fu. ArlJ       ILAAEVGALLVIKYRMPEDPIWAEAKVRTPRMVKIRKALILSILLVPIVGLVYFLFIRPR     297
                  *: .*   :  **  :*:**** :: ::*   :***: :*:: :*  :  *. :*

O. meri. ArlJ     TRIPFYFDVAIVITPFAWPGIVGSREEKKITKKDDMFGSFIRSLAGSASARGNMIIDALK     349
P. fu. ArlJ       FSIPTPFVVAIILTPLAYVGNVVRKEEGVIFRKDENFPAFIRSLSSSLAASGASLLLVLK     357
                    **  * ***::**:*: * *  :**  * :**: * :*****:.* :* *  :: .**

O. meri. ArlJ     SIVLHDFGSLTADIRNLYKRLTYRISNKEAWRNFSAETGSHLIEVFSESFMESVDLGSDA     409
P. fu. ArlJ       YLSAHDFGTLTEDIKALYRRLAIRVDQDRAWDYFIAETGSWLVGMFSEIFRESLRLGAEP     417
                   :  ****:** **: **:**: *:.:..**  * ***** *: :*** * **: **::

O. meri. ArlJ     EKAGMVVADNFDKVIRLRKRRHSSVASYVGVIYGITGGLAFSLAISYGVLEIISKVFSTL     469
P. fu. ArlJ       DYVGKVISRNFERIVRLRRKRQQSISNFIGIILGLTGAFAFSLAASFQVAVSINNLFSQL     477
                  : .* *:: **:::***::*::*:.*::.::*:* *:**.:***** *: *   *.::** *

O. meri. ArlJ     DVSSLQDFGIFVAQPPSELFIIEIFIVAILFLHSFVAGTALEIADGGRVAHSLHHAVIMI     529
P. fu. ArlJ       QVPTEYIGEIIHIIPPSGMQLLTISMLTLMILHSFLSAVAIKVADGGHLLVSLYYFVILL     537
                  :* :     *:   *** : :: * ::::::****::..*:::****::  **:: **::

O. meri. ArlJ     WIVSFVIYGTLQVVVLMLGGGL---------                                  551
P. fu. ArlJ       WVFAAGMYVGQLLMARFMNFGTTEMILLLVR                                  568
                  *:.:  :*    ::. ::. *
```

Supplementary Figure 13: Sequence alignments between archaellum machinery genes of *O. meridianum* and *P. furiosus* [4].

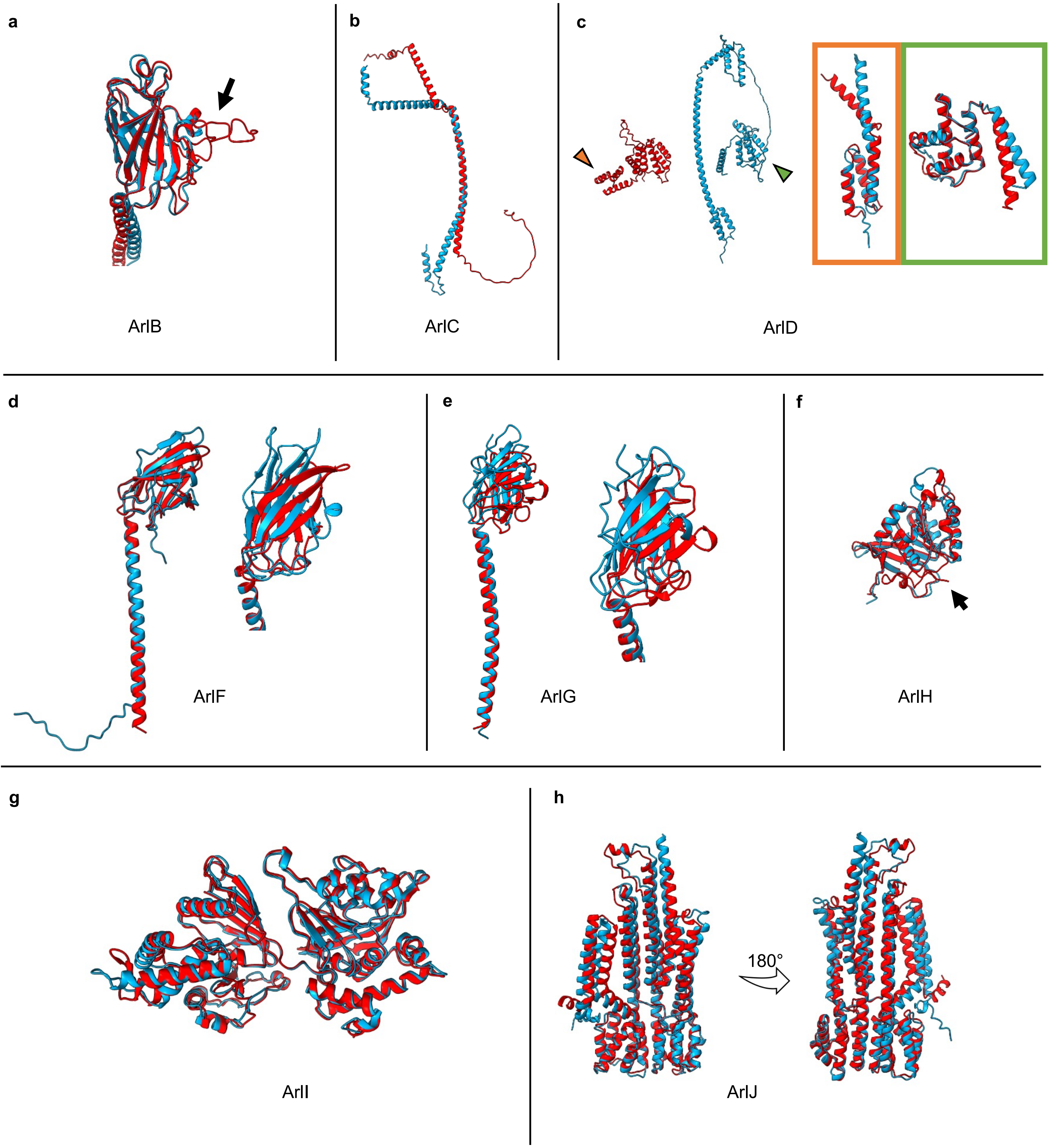

Supplementary Figure 14: Alphafold2 predictions suggest structural conservation between archaellum machinery proteins of *O. meridianum* and *P. furiosus*. Alphafold2 [2] predictions of archaellum machinery genes from *O. meridianum* (red) and *P. furiosus* (blue). Superimpositions of **a,** major archaellins show that *O. meridianum* has a unique extended loop (black arrow). **b, c** superimposition of ArlC (b) and ArlD (c) proteins (proposed cytoplasmic stator subunits). Due to the flexibility of these proteins, the N (orange box) and C (green box) termini were superimposed separately. **d**,**e,** superimposition of ArlF (d) and ArlG (e) (both proposed periplasmic stator and S-layer integration subunits). **f,** superimposition of the proposed regulator ArlH (**f**), the ATPase ArlI (**g**) and the membrane platform protein ArlJ (**h**).

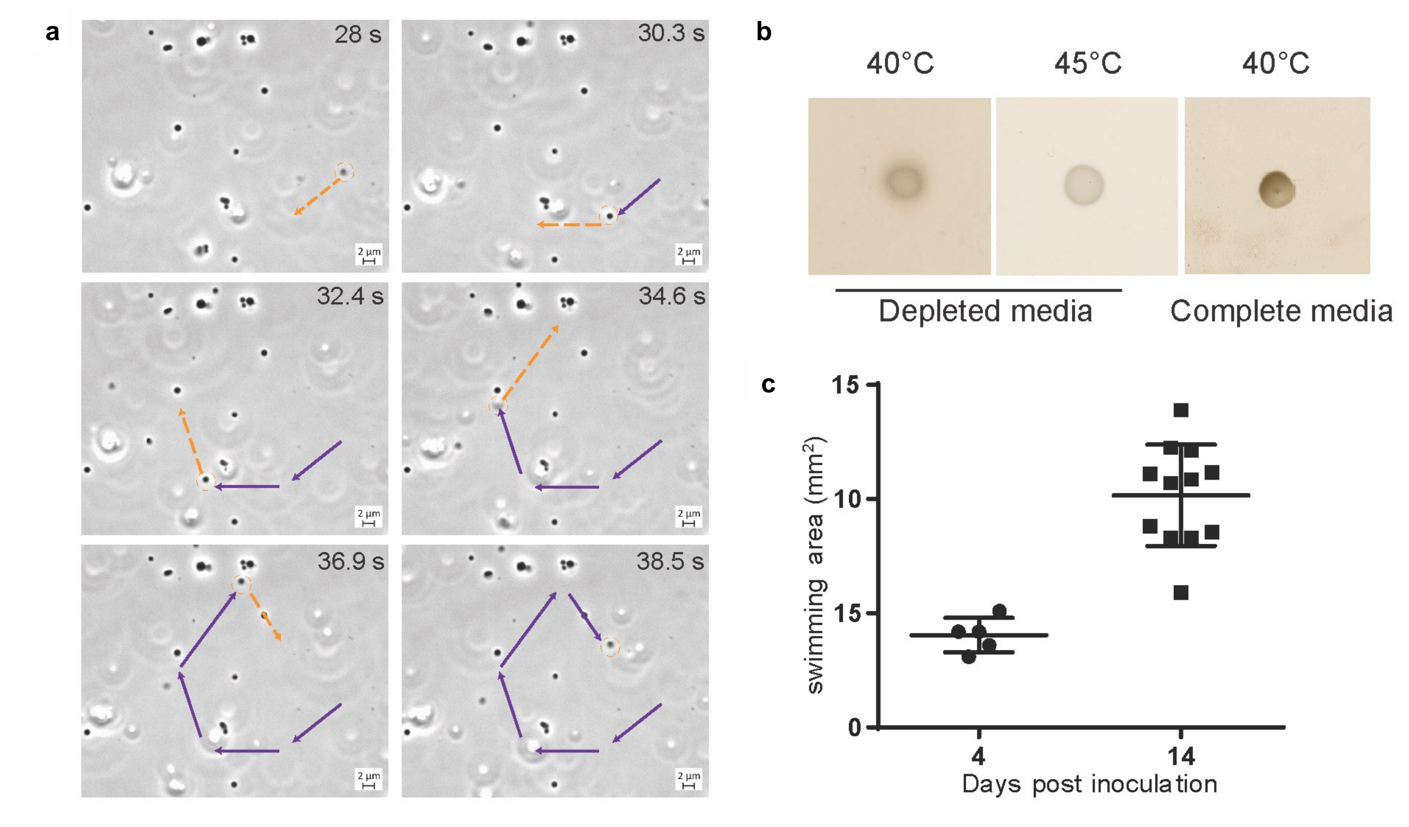

Supplementary Figure 15: *O. meridianum* is a motile species

a, Live cell microscopy of swimming *O. meridianum* cells at 55°C. One cell trajectory is indicated as example: Orange dotted arrows indicate future movements and purple arrows past movements of the cell indicated in the orange dotted circle. Time points are indicated in the upper corner. Scale bar 2 µm. **b** and **c**, motility assay of *O. meridianum* in semi-solid gelrite plates at different temperatures. **b,** representative images of swimming halos. **c,** swimming area in relation to days after inoculation in depleted media plates at 40°C.

```
O. meri. ArlF       ----MGFSYVAAVAILLSSSLIFFGIIYSDYVHSETQLSNAQNDQNRQMYDYINSKVKIT  56
T. aci. ArlF        MTSCMGFSYTVAAVIMLSSTLIFFGIVYTSYVQSNENIANANQKLVKSIYDLENTHVSIT  60
                        *****..*..*:***:******:*:.**:*: :::**::.  :.:**  *::*.**

O. meri.. ArlF       GYDVSPYNSVYNVTINMTNTGSISLDLMNSNLLLNGSLENFSYSAEYLLPMANGSVSFQT 116
T. aci. ArlF        GYYYNSSSSLFI--VNLTNNGSQVFNMSMANVLINGTMVKFNVSGPYLFPLQAVSISFKE  118
                    **  .  .*::   :*:**.**  :::  :*:*:**:: :*. *. **:*:   *:**:

O. meri.. ArlF       TAGNYQVEIAFNTGYNVITEVKV    139
T. aci. ArlF        PAGTYSLEIVMPDGYEIFREVVS     141
                     **.*.:**.:  **::: **
```

```
O. meri. ArlF       MGFSYVAAVAILLSSSLIFFGIIYSDYVHSETQLSNAQNDQNRQMYDYINSKVKITGYDV  60
T. vol. ArlF        MGFSYTVAAVIMLSSTLIFFGIVYSSYVQSNTSITNANQKLIKNIYNLENTKIEIVSYYY  60
                    *****..*..*:***:******:**.**:*:*.::**::.  :::*:  *:*::*..*

O. meri.. ArlF       SPYNSVYNVTINMTNTGSISLDLMNSNLLLNGSLENFSYSAEYLLPMANGSVSFQTTAGN 120
T. vol. ArlF        NSSSSTF--YVNVTDNGSTSFNLSESNVILNGTLISFNESNQYIFPLETITISFKASPGI  118
                    .  .*.:   :*:*:.** *::* :**::***:* .*. * :*::*: . ::**::: *

O. meri.. ArlF       YQVEIAFNTGYNVITEVKV 139
T. vol. ArlF        YSFELVMPDGYEVFKEVKS  137
                    *..*:.:  **:*:.***
```

```
O. meri. ArlG       ------------------------------------------------------------  0
T. aci. ArlG        MRYAAGQGNGYAVVSAQCFIYHYFSDLAWVENAGISWGLKLNVRSRDYIAVYEYGYRCIG  60

O. meri. ArlG       -----MASI----ATSEMIMFIATMVLT-----ASVVGVLGGETLHLSNSLTDSSASAST  46
T. aci. ArlG        QGKGHASRILYVKYITTIIRYLCRIVYYREVLLYHLRGFLR-RCLHAVHKME--------  111
                          : *      : :* ::. :*         : *.*  . **  :.:

O. meri. ArlG       QIQTDFKIINDPSQIPKDKGYVFYVKNTGQVGFDFTNNTVSTIINGSIVTGGYISYITPA  106
T. aci. ArlG        ------RLIRQH--SHRRRGYEQCYDEEDQFRYCCRSHQDTTSLNIS-------------  150
                          ::*.:     : :**    .: .*. :   .:  :* :* *

O. meri. ArlG       GSNGELMPSQVGTIVVNVTLSPGYHTIVITLNDGVTQQMVFEID    150
T. aci. ArlG        --------------------------------------------    150
```

```
O. meri.. ArlG       MASIATSEMIMFIATMVLTASVVGVLGGETLHLSNSLTDSSASASTQIQTDFKIINDPSQ 60
T. vol. ArlG        MASTAVSELVFFIVTLLITASAVAVLSDQTIHIVYGMQSSSQRTTDMIQENFAIINNPDQ  60
                    *** *.**:::**.*:::***.*.**..:*:*:  .: .**  ::  ** :* ***:*.*

O. meri.. ArlG       IPKDKGYVFYVKNTGQVGFDFTNNTVSTIINGSIVTGGYISYITPAGSNGELMPSQVGTI 120
T. vol. ArlG        IPYDNGYVFYIKNTGSVGFAFDNTSISVLVDGSAVTGKYVKFISLEN-SSILVPGQVGEI  119
                    ** *:*****:****.*** * *.::*.:::** *** *:.:*:  . .. *:*.*** *

O. meri.. ArlG       VVNVTLSPGYHTIVITLNDGVTQQMVFEID    150
T. vol. ArlG        VVNETL-SGYNSITISLGTGVSHTFNFEV-     147
                    *** **  **::*.*:*. **:: : **:
```

Supplementary Figure 16: Sequence alignment of ArlF and ArlG

Sequence alignments between *O. meridianum* and *T. acidophilum* ArlF; *O. meridianum* and *T. volcanium* ArlF; *O. meridianum* and *T. acidophilum* ArlG; and *O. meridianum* and *T. volcanium* ArlG.

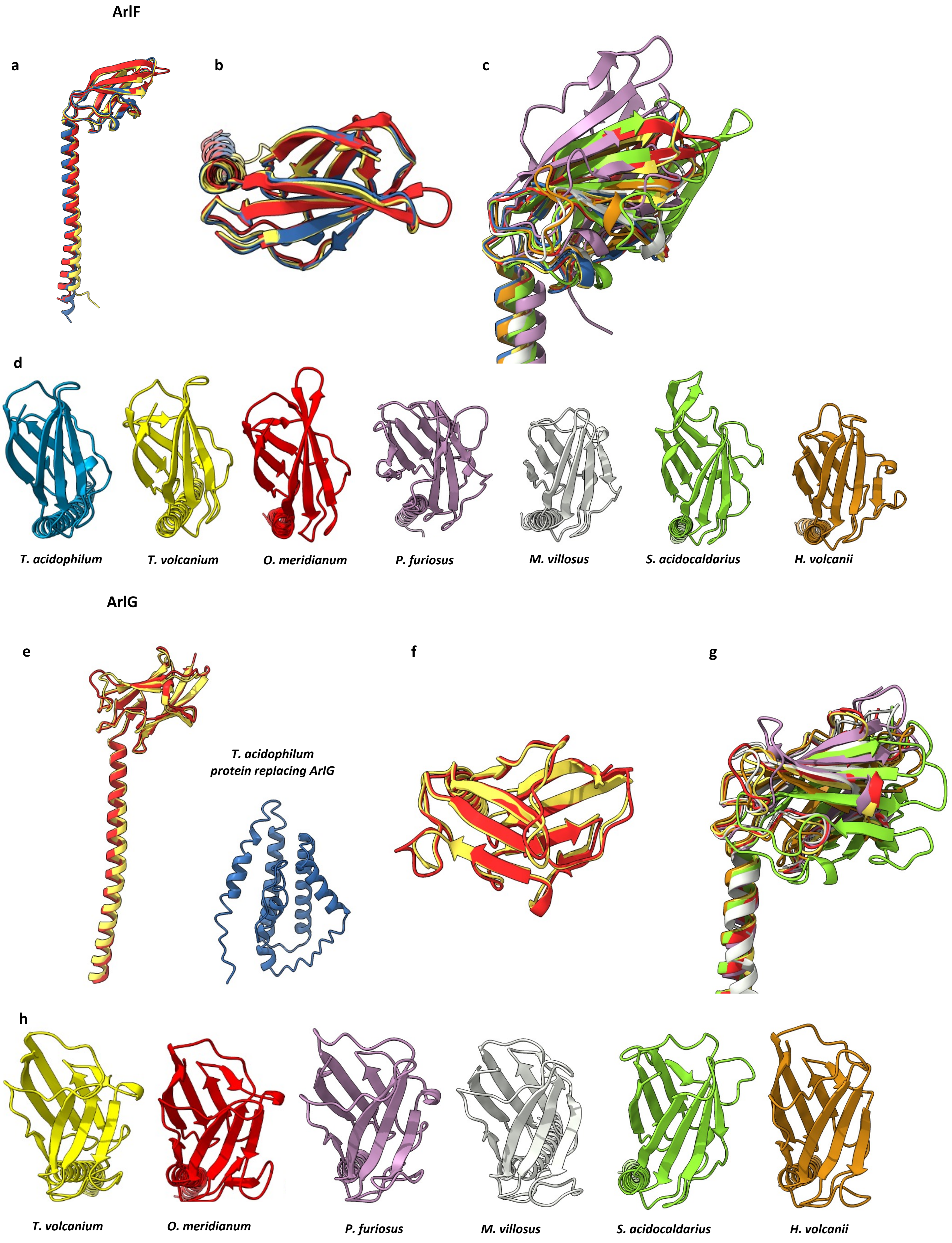
ArlF
a
b
c
d
T. acidophilum
T. volcanium
O. meridianum
P. furiosus
M. villosus
S. acidocaldarius
H. volcanii
ArlG
e
T. acidophilum protein replacing ArlG
f
g
h
T. volcanium
O. meridianum
P. furiosus
M. villosus
S. acidocaldarius
H. volcanii

Supplementary Figure 17: Structural conservation of ArlF and ArlG

Superimposed Alphafold2 [2] predictions of ArlF from *O. meridianum* (red) and other thermoplasmata species, *T. acidophilum* (blue) and *T. volcanium* (yellow) in side view **(a)** and top view **(b)**. Superimposed Alphafold2 [2] predictions (**c**) and side-by-side comparisons (**d**) of ArlF from *O. meridianum* (red) and the distantly related archaea *P. furiosus* (pink), *M.villosus* (white), *S. acidocaldarius* (green) and *H. volcanii* (orange). **e**,**f**, superimposed Alphafold2 [2] predictions of ArlG from *O. meridianum* (red) and related *thermoplasmata* species, *T. acidophilum* (blue) and *T. volcanium* (yellow) side view **(e)** and top view, **(f).** The *T. acidophilum* protein replacing ArlG is shown in blue. Superimposed Alphafold2 [2] predictions (**g**) and side-by-side comparisons (**h**) of ArlG from *O. meridianum* (red) and distantly related *P. furiosus* (pink), *M.villosus* (white), *S. acidocaldarius* (green), *H. volcanii* (orange).

Supplementary Table 1 – CryoEM and model building statistics.

	***O. meridianum* archaellum**<br>**(EMDB EMD-19168)**<br>**(PDB 8RH5)**	***C. divulgatum* filament**<br>**(EMDB EMD-19112)**<br>**(PDB 8REY)**
Data collection and processing		
Magnification	130k	130k
Voltage (kV)	200	200
Electron exposure (e–/Å²)	51.45	51.40
Defocus range (μm)	-0.8 to -2.0	-0.8 to -2.0
Pixel size (Å)	1.05 (0.525)	1.05 (0.525)
Symmetry imposed	Twist: 107.92° Rise: 5.6	Twist: 106.05° Rise: 5.19
Initial particle images (no.)	1,382,613	1,411,881
Final particle images (no.)	1,374,113	645,487
Map resolution (Å)	2.52	2.61
FSC threshold	0.143	0.143
Map resolution range (Å)	100-2.54	100-2.61
Resolution (Å) (map/model; FSC = 0.5)	2.7	2.7
Refinement		
Initial model used (PDB code)	Ab initio	Ab initio
Model resolution (FSC = 0.50/0.143 Å)	2.83/2.52	2.9/2.6
Model refinement resolution (Å)	2.52	2.61
Map sharpening B factor (Å²)	0	0
Model composition		
Number of protein monomers	31	36
Non-hydrogen atoms	67,485	40,140
Protein residues	7,590	4,860
Glycans	1,023	504
B factors (Å²)		
Protein	95.6	93.5
Glycan	176.6	211.3
R.m.s. deviations		
Bond lengths (Å)	0.008	0.008
Bond angles (°)	1.545	1.376
Validation		
MolProbity score	0.5	0.72
Clashscore	0.01	0.46
Poor rotamers (%)	0.0	0.0
Ramachandran plot		
Favored (%)	98.68	97.74
Allowed (%)	1.32	2.26
Disallowed (%)	0.0	0.0

Supplementary Table 2: Comparison of archaellins with experimentally determined structure. Growth temperature, pH values and number of N-glycosylation sites for 6 species.

Species	Optimum pH	Optimum Temperature (°C)	Filament Type	N-Glycosylations Sites Per Monomer	PDB Code
Pyrococcus furiosus	7.0	100	Archaellum	5	5O4U[4]
Methanospirillum hungatei	7.0	34	Archaellum	1	5TFY[5]
Halobacterium salinarium	7.0	37	Archaellum	3	9EQ7[6]
Methanocaldococcus villosus	6.5	80	Archaellum	ArB1: 3<br>ArlB2: 5	7OFQ[7]
Sulfolobus islandicus	2.7	80	Archaellum	3	8CWM[8]
Sulfolobus acidocaldarius	2.0	80	Archaellum	6	8QX4[9]
Oxyplasma meridianum	1.0	40	Archaellum	9 (8)	8RH5

SupplementaryTable 3: Comparison of pilins with experimentally determined structure. Growth temperature, pH values and number of N-glycosylation sites for 4 species.

Species	Optimum pH	Optimum Temperature (°C)	Filament Type	N-Glycosylations Sites Per Monomer	PDB Code
Sulfolobus solfataricus	3.0	75	T4P (Aap)	0	6W8X[10]
Sulfolobus islandicus	2.7	80	T4P (Aap)	0	6NAV[11]
Sulfolobus acidocaldarius	2.0	80	T4P (Aap)	4	8Q30[12]
Cuniculiplasma divulgatum	1.0	40	T4P (CdiP)	2	8REY

Supplementary references

1. Jamali, K., Käll, L., Zhang, R. et al. Automated model building and protein identification in cryo-EM maps. Nature 2024; 628:450–457
2. Bryant P, Pozzati G, Elofsson A. Improved prediction of protein-protein interactions using AlphaFold2. Nat Comms 2022;13:1–11.
3. Chen L, Brügger K, Skovgaard M, Redder P, et al. The Genome of *Sulfolobus acidocaldarius*, a Model Organism of the *Crenarchaeota*. J Bacteriol 2005; 187(14):4992–4999
4. Daum B, Vonck J, Bellack A, et al. Structure and *in situ* organisation of the *Pyrococcus furiosus* archaellum machinery. Elife 2017; 6:e27470.
5. Poweleit N, Ge P, Nguyen HH et al. CryoEM structure of the *Methanospirillum hungatei* archaellum reveals structural features distinct from the bacterial flagellum and type IV pilus. Nat Micro 2016; 2:1–12.
6. Sofer S, Vershinin Z, Mashni L et al. Perturbed N-glycosylation of *Halobacterium salinarum* archaellum filaments leads to filament bundling and compromised cell motility. Nat Comms 2024;15:1–13.
7. Gambelli L, Isupov MN, Conners R et al. An archaellum filament composed of two alternating subunits. Nat Comms 2022; 13:1–11.
8. Kreutzberger MAB, Sonani RR, Liu J et al. Convergent evolution in the supercoiling of prokaryotic flagellar filaments. Cell 2022; 185:3487-500
9. Gaines MC, Isupov MN, McLaren M et al. Towards a molecular picture of the archaeal cell surface. Nat Comms 2024; 15:1–16.
10. Wang F, Baquero DP, Su Z et al. The structures of two archaeal type IV pili illuminate evolutionary relationships. Nat Comms 2020; 11:1–10.
11. Wang F, Cvirkaite-Krupovic V, Kreutzberger MAB et al. An extensively glycosylated archaeal pilus survives extreme conditions. Nat Micro 2019; 4:1401–10.
12. Gaines MC, Sivabalasarma S, Isupov MN et al. CryoEM reveals the structure of an archaeal pilus involved in twitching motility. Nat Commun 2024; 15:1–13.
